# GRAG-Net: A Gated Residual Autoregressive–Exogenous GRU Soft Sensor for Dynamic SO_2_ Estimation in Sulfur Recovery Units

**DOI:** 10.3390/s26196168

**Published:** 2026-09-29

**Authors:** Maha Mesfer Alghamdi

**Affiliations:** Department of Computer Science and Engineering, College of Applied Studies, King Saud University, Riyadh 11451, Saudi Arabia; mmesfer@ksu.edu.sa

**Keywords:** sulfur recovery unit, sulfur dioxide, dynamic soft sensor, gated recurrent unit, autoregressive–exogenous modeling, residual learning, adaptive fusion, time series regression

## Abstract

Accurate sulfur dioxide (SO_2_) estimation is important for sulfur recovery unit (SRU) monitoring when reliable one-step information is required between analyzer observations. This study proposes the Gated Residual Autoregressive–Exogenous GRU Network (GRAG-Net), which separates multivariate process dynamics from strictly causal SO_2_ history, predicts branch-specific residual corrections relative to the latest measured target, and combines them through a sample-dependent gate. The model was evaluated on 10,081 chronologically ordered industrial observations using three expanding future test periods and five final random seeds. Comparators included persistence, linear extrapolation, absolute and residual classical regressors, process-only and autoregressive GRUs, a joint GRU, LSTM–ARX, TCN–ARX, fixed fusion, and global gating. Across 15 descriptive fold–seed evaluations, GRAG-Net achieved MAE 0.00315±0.00044 mole%, RMSE 0.00790±0.00064 mole%, and $R^{2}=0.97753\pm 0.00331$. Pooled seed-ensemble MAE and RMSE were 0.00291 and 0.00766 mole%, reductions of 32.5% and 18.0% relative to AR–GRU. Independently trained ablations identified residual reconstruction and sample-dependent fusion as the main architectural contributors, while dependence-aware bootstrap analysis, causal gate diagnostics, lag analysis, and cross-fold/cross-seed SHAP stability supported robust and interpretable one-step soft sensing.

## 1. Introduction

Sulfur dioxide (SO_2_) is an important atmospheric pollutant associated with industrial combustion, mineral processing, petroleum refining, and other sulfur-intensive operations. Following emission, SO_2_ participates in atmospheric oxidation and aerosol-formation processes that contribute to acid deposition, visibility degradation, and fine-particle formation [1]. Exposure to polluted air is also associated with a substantial global burden of respiratory and cardiovascular disease [2]. In petroleum refineries, accurate monitoring of sulfur compounds is therefore required not only for environmental compliance, but also for maintaining stable process operation, detecting abnormal conditions, and supporting timely corrective action.

Sulfur recovery units (SRUs) convert hydrogen-sulfide-rich acid gases into elemental sulfur, commonly through a thermal stage followed by catalytic Claus conversion and downstream tail-gas treatment. The residual H_2_S and SO_2_ concentrations depend on interacting feed conditions, airflow distribution, reaction conditions, catalyst state, and process inertia [3,4]. These relationships are nonlinear and time-dependent, and the effects of an operating adjustment may not be observed immediately because of transport delays and internal process dynamics. Hardware analyzers provide the reference gas-concentration measurements, but their readings can be delayed and their operation requires calibration and maintenance. Data-driven soft sensors can therefore provide complementary real-time estimates for monitoring and diagnostic support [3,5]. In the deployment setting considered here, the hardware analyzer remains the reference measurement source: GRAG-Net estimates the current SO_2_ concentration from process measurements available through time *t* and measured analyzer history available only through t−1. The soft sensor is therefore intended to complement the analyzer by providing a model-based one-step estimate between successive validated measurements or during measurement delay; it is not intended to permanently replace the physical analyzer. Continued analyzer measurements remain necessary for reference supervision, performance monitoring, and provision of the causal autoregressive history. Sustained analyzer-outage forecasting, in which predictions must recursively replace missing analyzer values, is outside the scope of this study.

A soft sensor estimates a difficult, delayed, or costly quality variable from routinely measured process information. Data-driven soft sensors are particularly attractive when sufficiently accurate first-principles models are unavailable or too expensive for online use [6,7,8]. Their practical development, however, is complicated by correlated measurements, nonlinear behavior, temporal dependence, measurement imperfections, limited target observations, and changes in operating conditions. Model reliability can deteriorate under process drift, catalyst aging, equipment fouling, raw-material variation, and transitions between operating regimes [9,10]. These characteristics make both dynamic representation and leakage-aware temporal validation essential for industrial soft sensing.

Research on the benchmark SRU process reflects the broader evolution of the field. Early studies considered neural soft analyzers and linear or nonlinear autoregressive models with exogenous inputs [3,11]. Bidar et al. introduced a multi-state-dependent parameter formulation to represent changes in operating state [12], whereas Yuan et al. developed a stacked isomorphic autoencoder for nonlinear feature extraction [13]. Dynamic sequence modeling was later investigated using echo-state networks and recurrent neural networks [14,15]. More recent work has addressed transferability and adaptation through transfer–incremental learning [16], as well as limited labeled data and nonlinear target decomposition through an ISSA–VMD–ESN framework [17]. Collectively, these studies demonstrate the importance of temporal modeling, nonlinear representation, and adaptation in SRU soft sensing.

Dedicated SO_2_ modeling has also progressed toward increasingly complex temporal architectures. Thameem et al. compared conventional machine learning methods, multilayer perceptrons, one-dimensional convolutional networks, LSTMs, and a CNN–LSTM encoder–decoder for multi-horizon SO_2_ forecasting in an industrial SRU [4]. Sun et al. subsequently proposed a multistage framework combining variational mode decomposition, CEEMDAN, entropy-guided component reconstruction, an enhanced PatchTST model, and metaheuristic hyperparameter optimization for SRU sulfur gas forecasting [18]. Li and Hao developed a dual-path dynamic soft sensor in which a TCN–Transformer processes auxiliary process variables and a GRU processes historical target values; the two learned representations are combined by summation before final prediction [19]. These recent studies confirm that process-variable histories and target histories provide complementary information. They also expose a remaining question: how should their relative contributions be adjusted when the local operating condition changes?

Existing SRU models generally improve the overall representation through deeper encoders, decomposition, transfer learning, or fixed feature fusion. However, three limitations remain relevant to the present problem. First, process measurements and historical SO_2_ values represent physically different predictive mechanisms. The latter primarily describe persistence and process inertia, whereas the former provide evidence of process-induced deviation. Embedding both sources in one representation, or combining them using a fixed operation, does not explicitly preserve this distinction. Second, the reliability of the two sources is sample dependent: target persistence can be highly effective under quasi-steady operation but less responsive during rapid transitions, while exogenous airflow information may become more valuable when the process state changes. Third, average test metrics alone do not reveal whether an adaptive fusion mechanism responds meaningfully to branch disagreement, changing operating conditions, or high-change target regimes. In addition, performance estimation for nonstationary time series should preserve temporal order and should separate model selection from evaluation on future observations [20].

To address these limitations, this study proposes the *Gated Residual Autoregressive–Exogenous GRU Network* (GRAG-Net) for one-step dynamic SO_2_ soft sensing. GRAG-Net contains two independently parameterized recurrent branches. The process branch encodes a multivariate history of five routinely measured airflow variables, whereas the autoregressive branch encodes only the strictly causal SO_2_ history. Rather than predicting the absolute concentration independently, each branch estimates a residual correction relative to the most recent available analyzer value. A context-dependent gate then combines the two corrections according to

(1) $${\hat{y}}_{t}=y_{t-1}+g_{t}\Delta {\hat{y}}_{t}^{x}+\left(1-g_{t}\right)\Delta {\hat{y}}_{t}^{y},\;0\leq g_{t}\leq 1,$$

where $\Delta {\hat{y}}_{t}^{x}$ and $\Delta {\hat{y}}_{t}^{y}$ denote the process-driven and autoregressive residual corrections, respectively. The gate is conditioned on the projected features of both branches, the latest SO_2_ value, the disagreement between the two proposed corrections, and the most recent change in the process variables. Thus, GRAG-Net performs sample-wise soft routing between process-driven and history-driven information instead of applying a global fusion weight. A robust Huber objective is used to reduce excessive sensitivity to isolated large errors [21].

The main contributions of this study are as follows:

1. A dual-branch residual architecture is developed in which exogenous process dynamics and autoregressive SO_2_ persistence are encoded separately and fused through a sample-dependent reliability gate.
2. The estimation task is defined explicitly as causal rolling one-step soft sensing: process measurements are available through time *t*, measured SO_2_ history is available only through t−1, and neither the current target nor future targets are supplied to the model. Sustained analyzer-outage forecasting is therefore outside the scope of the present study.
3. The comparative evaluation is expanded to include persistence and linear extrapolation, absolute and residual classical ARX regressors, strict process-only and autoregressive GRUs, a joint GRU, LSTM–ARX, TCN–ARX, equal-weight fusion, and a global-gate control, in addition to the proposed dynamic gate.
4. Architectural claims are tested using independently trained ablations of the residual formulation, fusion mechanism, branch supervision, gate-balance regularization, disagreement input, and process-change input. Chronological folds are treated as the primary evaluation periods, and paired moving-block bootstrap intervals are used to quantify uncertainty in sample-level error differences.
5. The diagnostic analysis is made inference-aware: gate associations are evaluated against causal inputs, process–target delays are estimated using training-only lagged correlations, and SHAP explanations are aggregated across folds and seeds with explicit rank-stability analysis.

The experiments use a chronologically ordered industrial benchmark containing 10,081 observations, five airflow-related process measurements, and SO_2_ concentration as the target [3,22]. The task is formulated as causal one-step soft sensing: process measurements available at time *t* are combined with target observations available only through t−1. The current target and all future target values are excluded from the model input.

The remainder of this paper is organized as follows. Section 2 describes the dataset, causal sample construction, nested model development protocol, GRAG-Net architecture, benchmark models, evaluation criteria, and interpretability procedures. Section 3 presents the comparative and diagnostic results. Section 4 summarizes the principal findings, industrial implications, limitations, and future research directions.

## 2. Materials and Methods

This section describes the industrial sulfur recovery unit (SRU) dataset, the causal formulation of the dynamic soft-sensing task, the proposed Gated Residual Autoregressive–Exogenous GRU Network (GRAG-Net), the reference models, and the nested chronological model development protocol. Particular attention is given to preserving the temporal ordering of the plant records and to maintaining a strict separation between hyperparameter selection and final performance estimation. All preprocessing parameters, model configurations, and epoch-selection decisions were derived exclusively from the training information available within the corresponding chronological fold. The evaluation framework further considers chronological robustness, variability across random initializations, target-change regimes, residual behavior, and model interpretability.

### 2.1. Industrial Sulfur Recovery Process and Dataset

#### 2.1.1. Process Background and Data Source

The experimental data were obtained from a benchmark industrial sulfur recovery process associated with the fourth tail-gas line of an Italian refinery [3,22]. An SRU converts hydrogen-sulfide-rich acid gas streams into elemental sulfur, typically through a high-temperature thermal stage followed by catalytic Claus conversion and downstream tail-gas treatment. In the studied process, acid gas streams originating from the monoethanolamine (MEA) and sour-water-stripping (SWS) sections are combined with controlled air streams before entering the reaction system. The distribution of combustion and secondary air affects the stoichiometric balance of the sulfur-recovery reactions and, consequently, the residual sulfur-compound concentrations measured in the treated tail gas.

The mapping from routinely measured airflow variables to the resulting SO_2_ concentration is nonlinear and time dependent. Variations in acid gas loading, air allocation, reaction conditions, catalyst state, and downstream treatment do not necessarily affect the measured tail-gas composition instantaneously. Instead, transport delay, process inertia, nonlinear reaction behavior, and interactions among operating variables can produce delayed and regime-dependent responses. These characteristics make the dataset suitable for evaluating dynamic soft sensors that explicitly retain both recent process history and target persistence.

The observations were recorded at one-minute intervals. The original benchmark contains five process measurements and two sulfur gas outputs, H_2_S and SO_2_. The present study focuses exclusively on SO_2_ soft sensing; therefore, the analyzed data matrix contains the five process inputs and the SO_2_ target field, denoted by y2. Following column selection and numeric conversion, the retained dataset comprised

(2) N=10,081

complete observations in their original chronological order. No missing values were present in the retained variables, and no interpolation or imputation was therefore required. The five deposited process channels are supplied on a scaled [0,1] benchmark representation, whereas the SO_2_ target retains the concentration scale used in the originating SRU data and is reported in mole%. This interpretation is consistent with both the original refinery study and the recent public benchmark release [3,18]. The chronological order was preserved throughout the study, and no random shuffling was applied before constructing the training, validation, and test periods.

#### 2.1.2. Process Variables and Prediction Target

Let

(3) $$x_{t}={\left[x_{1,t},x_{2,t},x_{3,t},x_{4,t},x_{5,t}\right]}^{⊤}\in R^{5}$$

denote the process measurements available at sampling instant *t*. The corresponding target is the SO_2_ concentration $y_{t}$, reported in mole%.

The five airflow-related channels were treated as exogenous process measurements because they are routinely available from the plant instrumentation, whereas SO_2_ represents the difficult-to-measure quality variable estimated by the soft sensor. The process channels retain their benchmark scaling, while SO_2_ is reported in mole%. Physical variable names are retained in all tables, figures, lagged-feature labels, and SHAP explanations to preserve process-level interpretability.

#### 2.1.3. Descriptive and Distributional Characteristics

Table 1 reports descriptive statistics for the complete benchmark dataset. These statistics are provided only to characterize the benchmark; they were not used to preprocess validation or test observations. Fold-specific preprocessing parameters were estimated from the relevant training block, as described in Section 2.2.

The SO_2_ concentration is concentrated within a comparatively narrow operating range, with a mean of 0.1706, a median of 0.1665, and a standard deviation of 0.0569. Its third quartile is 0.1923, despite a maximum of 1.0 mole%, indicating that large concentration values are relatively infrequent. In contrast, the process measurements span broader and heterogeneous operating ranges. The median values of airflow in the SWS zone ($x_{4}$) and secondary airflow ($x_{5}$) are 0.7225 and 0.6953, respectively, showing that these channels operate near their upper benchmark-scaled regions during a substantial part of the observation period. Airflow in the MEA zone ($x_{3}$) is more centrally concentrated and exhibits the smallest process-variable standard deviation.

**Figure 1:**
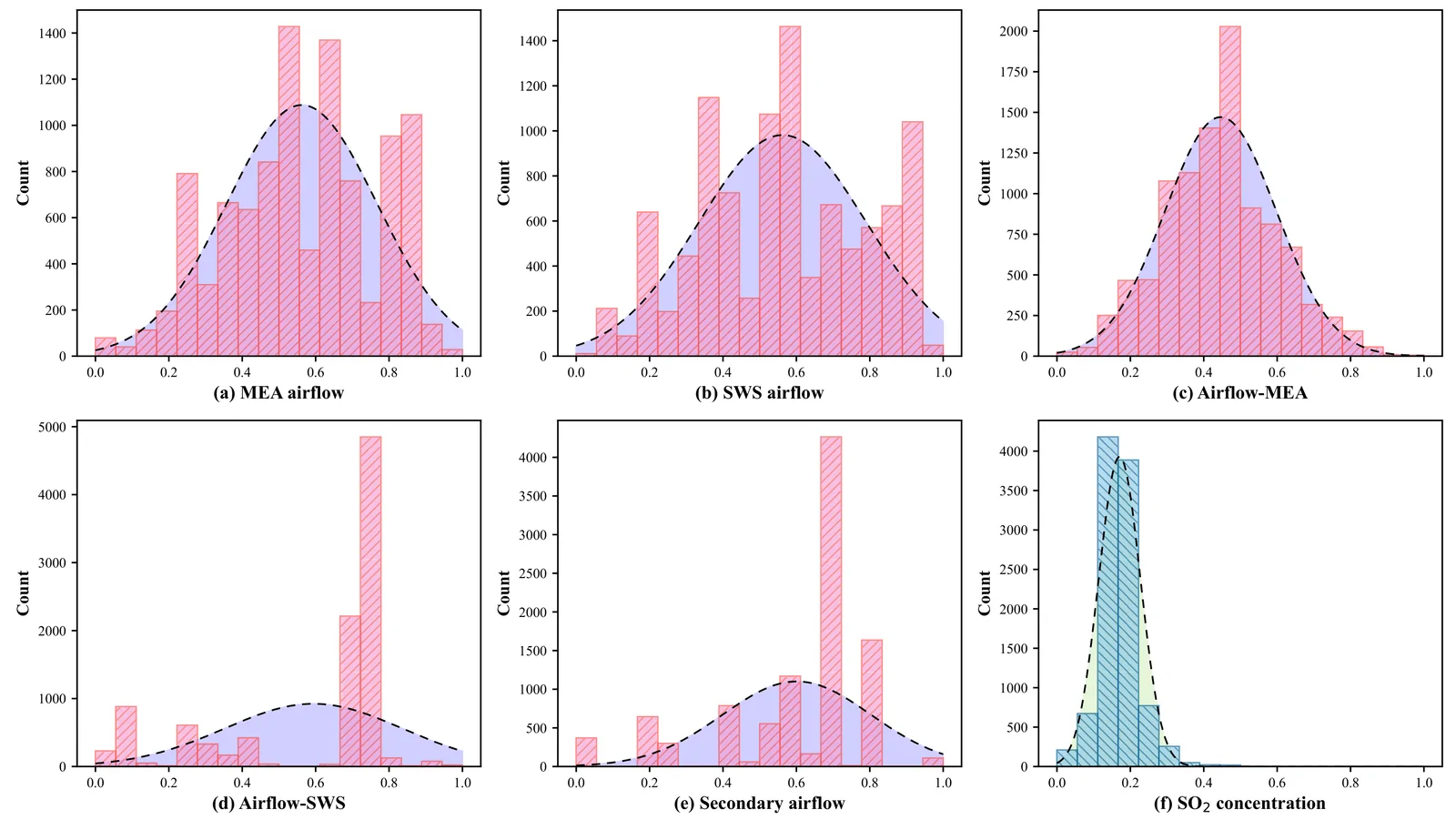
Histogram and density distributions of the five benchmark-scaled SRU process variables and SO_2_ concentration. The process measurements exhibit heterogeneous and partly multimodal operating distributions, whereas the target is concentrated within a narrow dominant range with a sparse upper tail.

The histogram and density plots in Figure 1 show that several process variables have heterogeneous and visibly multimodal distributions. These modes indicate recurring operating regions rather than one stationary Gaussian regime. The SO_2_ target has a narrow dominant distribution with a sparse upper tail. This imbalance is relevant because a model may achieve a low average error in the dominant quasi-steady region while remaining less accurate during rare concentration excursions.

#### 2.1.4. Correlation Structure and Target Persistence

**Figure 2:**
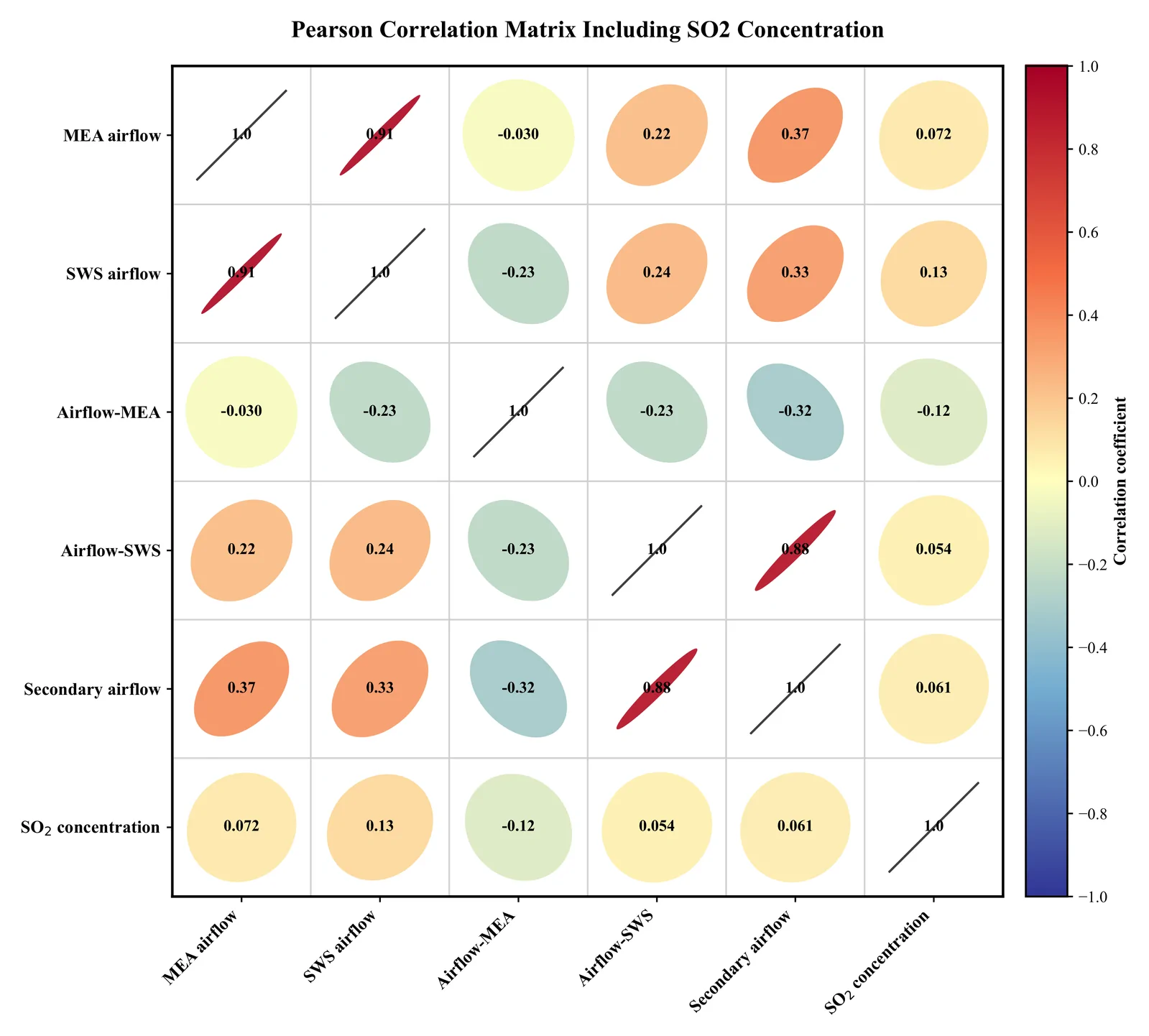
Pearson correlation ellipse matrix for the five SRU process variables and SO_2_ concentration. Ellipse orientation indicates the sign of the relationship, whereas increasing eccentricity denotes stronger linear dependence.

The Pearson correlation structure is shown in Figure 2. Strong dependence is present among selected process variables. The largest positive process-input correlations are observed between MEA airflow and SWS airflow,

(4) $$r(x_{1},x_{2})=0.913,$$

and between airflow in the SWS zone and secondary airflow,

(5) $$r(x_{4},x_{5})=0.876.$$

These values indicate substantial coupling and partial redundancy among the airflow measurements. In contrast, the zero-lag correlations between the five process variables and SO_2_ range approximately from −0.117 to 0.133.

The weak instantaneous input–target correlations do not imply that the process measurements are uninformative. Instead, they suggest that their effects on SO_2_ are delayed, nonlinear, dependent on interactions among variables, or conditional on the operating regime. This observation motivates the use of historical multivariate process windows rather than a static mapping based only on measurements recorded at the current instant.

In contrast to the weak zero-lag process–target associations, the SO_2_ sequence exhibits pronounced short-term persistence. The autocorrelation coefficients at lags one through five are

(6) $$\left[\rho_{y}\left(1\right),\rho_{y}\left(2\right),\rho_{y}\left(3\right),\rho_{y}\left(4\right),\rho_{y}\left(5\right)\right]=\left[0.9531,0.8487,0.7330,0.6273,0.5431\right].$$

The lag-one autocorrelation of 0.9531 confirms that the most recent SO_2_ observation is a strong local predictor and motivates its use as the persistence reference in GRAG-Net. However, the gradual decay across subsequent lags shows that the useful temporal information is not restricted to a single previous observation. This supports a sequence-based autoregressive encoder with a history length selected through nested hyperparameter optimization.

Taken together, the exploratory analysis identifies three properties that directly motivate the proposed methodology: (i) heterogeneous and partly multimodal process-variable distributions; (ii) weak instantaneous but potentially delayed and nonlinear process–target relationships; and (iii) strong short-term target persistence. These characteristics support the separation of process history and target history into two distinct temporal pathways before their contributions are fused dynamically.

### 2.2. Problem Formulation and Causal Sample Construction

#### 2.2.1. One-Step Dynamic Soft-Sensing Formulation

The objective is to estimate the current SO_2_ concentration $y_{t}$ from two information sources that are available at sampling instant *t*: (i) the recent history of the five routinely measured process variables and (ii) a strictly causal history of previously observed SO_2_ values. The task is therefore formulated as *one-step dynamic soft sensing*, rather than unconstrained recursive forecasting. Current process measurements are assumed to be available from the distributed control system at time *t*, whereas the target history channel terminates at t−1. Consequently, neither the current target $y_{t}$ nor any future target value is exposed to the model.

Let the instantaneous process vector be

(7) $$x_{t}={\left[x_{1,t},x_{2,t},x_{3,t},x_{4,t},x_{5,t}\right]}^{⊤}\in R^{5}.$$

A multivariate process-history window of length $L_{x}$ is defined as

(8) $$X_{t}={\left[x_{t-L_{x}+1},x_{t-L_{x}+2},\ldots ,x_{t}\right]}^{⊤}\in R^{L_{x}\times 5},$$

while the strictly causal SO_2_ history of length $L_{y}$ is

(9) $$Y_{t}={\left[y_{t-L_{y}},y_{t-L_{y}+1},\ldots ,y_{t-1}\right]}^{⊤}\in R^{L_{y}\times 1}.$$

The complete information set available for estimating $y_{t}$ is therefore

(10) $$I_{t}={\left\{x_{\tau }\right\}}_{\tau =t-L_{x}+1}^{t}\cup {\left\{y_{\tau }\right\}}_{\tau =t-L_{y}}^{t-1}.$$

The general soft-sensing mapping is expressed as

(11) $${\hat{y}}_{t}=F_{\theta }\left(X_{t},Y_{t}\right),$$

where $F_{\theta }\left(\cdot \right)$ denotes a nonlinear estimator with parameters θ. For GRAG-Net, $y_{t-1}$ is extracted from the final element of $Y_{t}$ and is used explicitly as both a gating-context variable and the residual reference in the final reconstruction. The proposed mapping can therefore be written more specifically as

(12) $${\hat{y}}_{t}=F_{\theta }\left(X_{t},Y_{t},y_{t-1}\right).$$

The two histories represent different predictive mechanisms. The process window $X_{t}$ describes the recent operating trajectory and provides exogenous evidence of process-driven changes, whereas $Y_{t}$ captures target persistence and process inertia. Their history lengths are allowed to differ because the useful temporal extent of the process variables need not match that of the target. Both $L_{x}$ and $L_{y}$ are therefore selected independently within the nested hyperparameter-optimization procedure.

#### 2.2.2. Fold-Specific Standardization

Although the process channels are supplied on a scaled benchmark representation and SO_2_ is retained in mole%, an additional z-score transformation was applied to improve numerical conditioning and to ensure that each model was developed using only the distributional information available in its training period. For process variable $x_{j}$,

(13) $${\tilde{x}}_{j,t}=\frac{x_{j,t}-\mu_{x_{j}}^{\mathrm{tr}}}{\sigma_{x_{j}}^{\mathrm{tr}}},\;j=1,\ldots ,5,$$

and the target was transformed as

(14) $${\tilde{y}}_{t}=\frac{y_{t}-\mu_{y}^{\mathrm{tr}}}{\sigma_{y}^{\mathrm{tr}}}.$$

Here, $\mu_{x_{j}}^{\mathrm{tr}}$, $\sigma_{x_{j}}^{\mathrm{tr}}$, $\mu_{y}^{\mathrm{tr}}$, and $\sigma_{y}^{\mathrm{tr}}$ were estimated exclusively from the training block available at the corresponding stage of the nested protocol. During inner hyperparameter optimization, the transformations were fitted only to the inner-training subset and were then applied unchanged to the inner-validation subset. After hyperparameter selection, new transformations were fitted to the complete outer-training block and applied unchanged to its held-out outer-test block.

The standardized history tensors are denoted by ${\tilde{X}}_{t}$ and ${\tilde{Y}}_{t}$. Neural-model training and prediction were performed in the standardized target domain. Each estimate was subsequently returned to the original benchmark scale using

(15) $${\hat{y}}_{t}=\sigma_{y}^{\mathrm{tr}}{\hat{\tilde{y}}}_{t}+\mu_{y}^{\mathrm{tr}},$$

before computing the reported evaluation metrics.

#### 2.2.3. Sliding-Window Sample Generation

Dynamic samples were constructed with a stride of one observation. Under the zero-based indexing used in the implementation, the first admissible target index for a given pair $\left(L_{x},L_{y}\right)$ is

(16) $$t_{\min}=\max\left(L_{x}-1,L_{y}\right),$$

which guarantees that both the complete process window in Equation (8) and the complete target history sequence in Equation (9) are available. For each admissible *t*, one supervised sample was formed as

(17) $$\left({\tilde{X}}_{t},{\tilde{Y}}_{t},{\tilde{y}}_{t}\right).$$

No downsampling, interpolation, or random reordering was applied during window construction.

The history lengths were treated as model hyperparameters rather than fixed a priori. The searched values were

(18) $$L_{x}\in \left\{16,32,64\right\},\;L_{y}\in \left\{4,8,16,32\right\}.$$

Because the records were sampled at one-minute intervals, these values correspond to process histories of 16, 32, or 64 min and target histories of 4, 8, 16, or 32 min.

#### 2.2.4. Causality and Chronological Boundary Handling

The chronological split boundaries were defined in terms of prediction indices, not isolated window matrices. At the beginning of a validation or test block, observations immediately preceding the boundary were retained as historical context when they belonged to the information set $I_{t}$. This treatment reflects online deployment: when the first sample of a future period is estimated, the recent process measurements and the valid analyzer observations recorded before that instant are already available.

For every prediction index *t*, the process sequence terminates at $x_{t}$ and the target sequence terminates at $y_{t-1}$. Thus, historical context may cross a training–validation or training–test boundary, but the prediction target itself and all future values remain excluded. The procedure does not create a gap between adjacent chronological blocks and does not discard the first $L_{x}$ or $L_{y}$ observations of each held-out period unnecessarily.

During outer-test evaluation, each prediction uses the measured SO_2_ history available through t−1. Predictions are not recursively fed back as substitutes for subsequent analyzer measurements. The reported task therefore represents a rolling one-step inferential setting in which a recent valid target history is available for every sample. This assumption should be distinguished from prolonged analyzer-outage forecasting, which would require recursive prediction and is outside the scope of the present study.

#### 2.2.5. Input Representation for the Baseline Models

The classical autoregressive–exogenous baselines cannot directly process two-dimensional sequences. Their standardized process and target histories were therefore flattened and concatenated:

(19) $$v_{t}=\left[\mathrm{vec}\left({\tilde{X}}_{t}\right);{\tilde{Y}}_{t}\right]\in R^{5L_{x}+L_{y}}.$$

The same causal information was consequently supplied to all learnable models, while only its representation differed. The support vector regressor and tree ensembles received $v_{t}$, whereas GRAG-Net retained the temporal organization of ${\tilde{X}}_{t}\in R^{L_{x}\times 5}$ and ${\tilde{Y}}_{t}\in R^{L_{y}\times 1}$ and processed the two histories through independent recurrent branches. This design ensures that comparative performance differences are not caused by unequal access to process or target history information.

### 2.3. Proposed GRAG-Net Architecture

#### 2.3.1. Design Rationale and Architectural Overview

The proposed Gated Residual Autoregressive–Exogenous GRU Network (GRAG-Net) is designed to model two complementary mechanisms governing one-step SO_2_ soft sensing. The first mechanism is the strong short-term persistence of the target, which makes the most recent valid SO_2_ observation an informative reference under quasi-steady operation. The second mechanism is the process-driven deviation from this recent trajectory, which is reflected in the evolving histories of the five airflow-related variables. Because the relative usefulness of these two information sources can change from one observation to another, GRAG-Net models them through independent temporal branches and combines their residual corrections using a sample-dependent reliability gate.

Let ${\tilde{X}}_{t}\in R^{L_{x}\times 5}$ and ${\tilde{Y}}_{t}\in R^{L_{y}\times 1}$ denote the standardized process and target history windows defined in Section 2.2. Rather than predicting the absolute target independently, each branch estimates a correction relative to the latest standardized SO_2_ observation ${\tilde{y}}_{t-1}$. The complete model is summarized by

(20) $$\Delta {\hat{\tilde{y}}}_{t}^{\;x}=f_{x}\left({\tilde{X}}_{t};\theta_{x}\right),$$

(21) $$\Delta {\hat{\tilde{y}}}_{t}^{\;y}=f_{y}\left({\tilde{Y}}_{t};\theta_{y}\right),$$

(22) $$g_{t}=f_{g}\left(c_{t};\theta_{g}\right),\;g_{t}\in \left[0,1\right],$$

(23) $${\hat{\tilde{y}}}_{t}={\tilde{y}}_{t-1}+g_{t}\Delta {\hat{\tilde{y}}}_{t}^{\;x}+\left(1-g_{t}\right)\Delta {\hat{\tilde{y}}}_{t}^{\;y}.$$

Here, $f_{x}\left(\cdot \right)$ and $f_{y}\left(\cdot \right)$ denote the process and autoregressive experts, respectively, while $f_{g}\left(\cdot \right)$ denotes the gating network. A value of $g_{t}$ approaching one gives greater influence to the process-driven correction, whereas a value approaching zero favors the autoregressive correction.

This residual formulation is particularly suitable for the studied target. The skip connection through ${\tilde{y}}_{t-1}$ explicitly preserves the strong persistence component, allowing the trainable branches to focus on the remaining change:

(24) $$\Delta {\tilde{y}}_{t}={\tilde{y}}_{t}-{\tilde{y}}_{t-1}.$$

Thus, GRAG-Net does not force either branch to repeatedly learn the near- identity mapping between consecutive target observations. Instead, both branches solve the more focused problem of estimating how the current target departs from its latest measured value.

**Figure 3:**
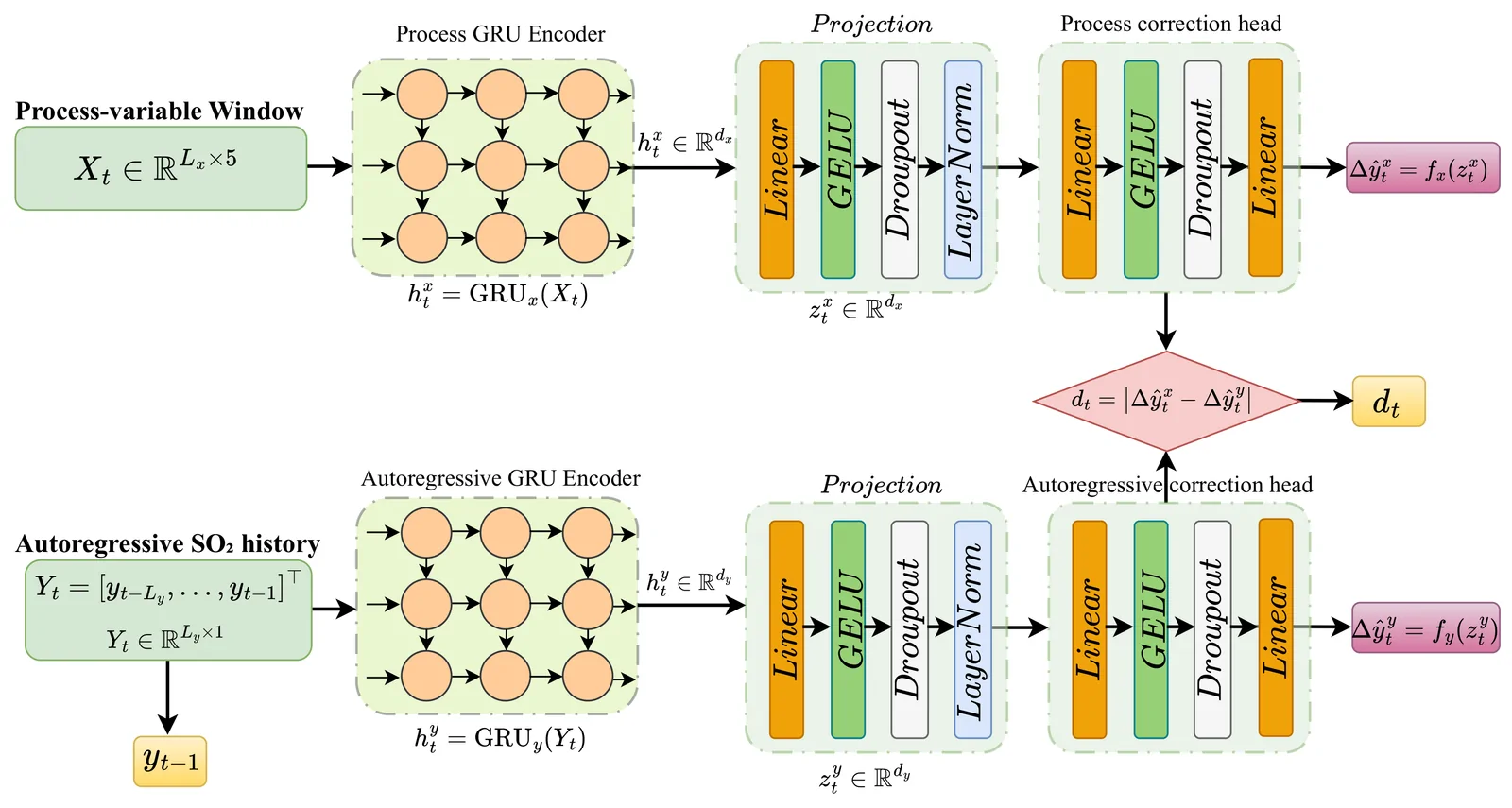
Dual-branch temporal encoding and residual-correction module of GRAG-Net. The process branch encodes the multivariate operating history, whereas the autoregressive branch encodes the strictly causal SO_2_ history. Each branch produces a dedicated residual correction relative to ${\tilde{y}}_{t-1}$, and the absolute difference between the two corrections defines the branch-disagreement statistic $d_{t}$.

**Figure 4:**
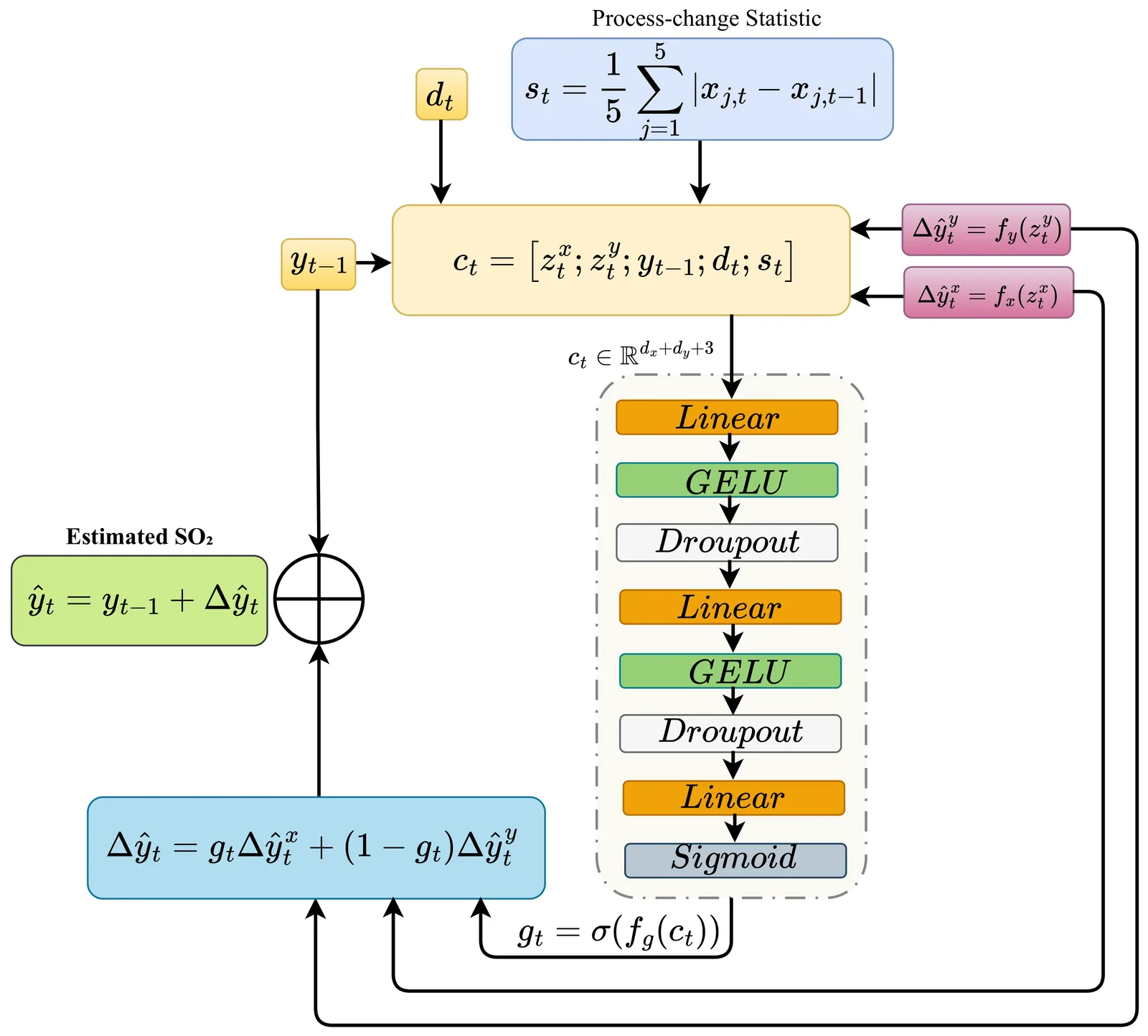
Context-dependent reliability gating and residual fusion in GRAG-Net. The projected process and autoregressive states, latest SO_2_ observation, branch disagreement, and recent process-change statistic form the gating context. The resulting process weight $g_{t}$ adaptively combines the two branch-specific residual corrections before addition to the persistence reference.

Figure 3 and Figure 4 present the architecture in two complementary parts. Figure 3 illustrates the independent temporal encoders, projection modules, and branch-specific correction heads, while Figure 4 shows the dynamic context, reliability-gate estimation, residual fusion, and final reconstruction. Sequence dimensions are denoted symbolically by $L_{x}$ and $L_{y}$.

#### 2.3.2. GRU-Based Temporal Encoding

A gated recurrent unit (GRU) was selected for both temporal branches because it provides explicit memory-control mechanisms with fewer trainable gates than an LSTM. For an input vector $u_{\tau }$ and previous hidden state $h_{\tau -1}$, a GRU computes

(25) $$r_{\tau }=\sigma \left(W_{r}u_{\tau }+U_{r}h_{\tau -1}+b_{r}\right),$$

(26) $$q_{\tau }=\sigma \left(W_{q}u_{\tau }+U_{q}h_{\tau -1}+b_{q}\right),$$

(27) $${\tilde{h}}_{\tau }=\tanh\left[W_{h}u_{\tau }+U_{h}\left(r_{\tau }⊙h_{\tau -1}\right)+b_{h}\right],$$

(28) $$h_{\tau }=\left(1-q_{\tau }\right)⊙{\tilde{h}}_{\tau }+q_{\tau }⊙h_{\tau -1},$$

where $r_{\tau }$ and $q_{\tau }$ are the reset and update gates, σ(·) denotes the logistic sigmoid, and ⊙ represents element-wise multiplication. Separate parameter sets are learned for the process and autoregressive GRUs; no recurrent weights are shared between the two branches.

#### 2.3.3. Process-Variable Expert

The process expert receives the standardized sequence ${\tilde{X}}_{t}$ and models nonlinear temporal interactions among the five process measurements. Its final GRU state is

(29) $$h_{t}^{x}={\mathrm{GRU}}_{x}\left({\tilde{X}}_{t}\right)\in R^{d_{x}},$$

where $d_{x}$ is the process-branch hidden dimension. The final state is passed through a projection block consisting of a linear transformation, Gaussian error linear unit (GELU), dropout, and layer normalization:

(30) $$z_{t}^{x}=\mathrm{LN}\left\{{\mathrm{Drop}}_{p}\left[\mathrm{GELU}\left(W_{p}^{x}h_{t}^{x}+b_{p}^{x}\right)\right]\right\}\in R^{d_{x}}.$$

Layer normalization stabilizes the latent scale before fusion, whereas dropout regularizes the projected representation.

A two-layer correction head maps $z_{t}^{x}$ to a scalar process-driven residual:

(31) $$a_{t}^{x}={\mathrm{Drop}}_{p}\left[\mathrm{GELU}\left(W_{x,1}z_{t}^{x}+b_{x,1}\right)\right],$$

(32) $$\Delta {\hat{\tilde{y}}}_{t}^{\;x}=W_{x,2}a_{t}^{x}+b_{x,2}.$$

The implemented head follows

(33) $$\mathrm{Linear}\left(d_{x},d_{x}/2\right)\to \mathrm{GELU}\to \mathrm{Dropout}\left(p\right)\to \mathrm{Linear}\left(d_{x}/2,1\right).$$

The resulting branch-level estimate is

(34) $${\hat{\tilde{y}}}_{t}^{\;x}={\tilde{y}}_{t-1}+\Delta {\hat{\tilde{y}}}_{t}^{\;x}.$$

Accordingly, the process expert learns the correction implied by the recent evolution of the airflow-related measurements.

#### 2.3.4. Autoregressive Expert

The autoregressive expert independently encodes the strictly causal target history. Its final hidden state is

(35) $$h_{t}^{y}={\mathrm{GRU}}_{y}\left({\tilde{Y}}_{t}\right)\in R^{d_{y}},$$

where $d_{y}$ is selected independently from $d_{x}$. The projected autoregressive representation is

(36) $$z_{t}^{y}=\mathrm{LN}\left\{{\mathrm{Drop}}_{p}\left[\mathrm{GELU}\left(W_{p}^{y}h_{t}^{y}+b_{p}^{y}\right)\right]\right\}\in R^{d_{y}}.$$

Its correction head is defined as

(37) $$a_{t}^{y}={\mathrm{Drop}}_{p}\left[\mathrm{GELU}\left(W_{y,1}z_{t}^{y}+b_{y,1}\right)\right],$$

(38) $$\Delta {\hat{\tilde{y}}}_{t}^{\;y}=W_{y,2}a_{t}^{y}+b_{y,2},$$

with layer sequence

(39) $$\mathrm{Linear}\left(d_{y},d_{y}/2\right)\to \mathrm{GELU}\to \mathrm{Dropout}\left(p\right)\to \mathrm{Linear}\left(d_{y}/2,1\right).$$

The corresponding branch-level estimate is

(40) $${\hat{\tilde{y}}}_{t}^{\;y}={\tilde{y}}_{t-1}+\Delta {\hat{\tilde{y}}}_{t}^{\;y}.$$

This branch provides a history-driven correction that exploits the shape, direction, and persistence of the recent SO_2_ trajectory rather than using only the single value ${\tilde{y}}_{t-1}$.

#### 2.3.5. Dynamic Context Construction

The reliability gate receives the learned representations of both experts together with two explicit dynamic descriptors. The first descriptor is the absolute disagreement between the proposed branch corrections:

(41) $$d_{t}=\left|\Delta {\hat{\tilde{y}}}_{t}^{\;x}-\Delta {\hat{\tilde{y}}}_{t}^{\;y}\right|.$$

A small value of $d_{t}$ indicates that the two experts recommend a similar departure from the persistence reference, whereas a large value indicates competition between their process-driven and history-driven explanations.

The second descriptor summarizes the most recent change in the standardized process measurements:

(42) $$s_{t}=\frac{1}{5}\sum_{j=1}^{5}\left|{\tilde{x}}_{j,t}-{\tilde{x}}_{j,t-1}\right|.$$

This statistic supplies the gate with a compact indicator of the local process dynamics. It is not used as a manually defined regime label and does not directly determine the gate value; instead, it is learned jointly with the latent branch representations.

The complete context vector is

(43) $$c_{t}={\left[{\left(z_{t}^{x}\right)}^{⊤},{\left(z_{t}^{y}\right)}^{⊤},{\tilde{y}}_{t-1},d_{t},s_{t}\right]}^{⊤}\in R^{d_{x}+d_{y}+3}.$$

Including ${\tilde{y}}_{t-1}$ allows the gating decision to depend on the current target level, while $d_{t}$ and $s_{t}$ expose the instantaneous degree of branch disagreement and process variation.

#### 2.3.6. Sample-Dependent Reliability Gate and Residual Fusion

The context vector is mapped to a scalar process-branch weight using a three-layer multilayer perceptron:

(44) $$v_{t,1}={\mathrm{Drop}}_{p}\left[\mathrm{GELU}\left(W_{g,1}c_{t}+b_{g,1}\right)\right]\in R^{d_{g}},$$

(45) $$v_{t,2}={\mathrm{Drop}}_{p}\left[\mathrm{GELU}\left(W_{g,2}v_{t,1}+b_{g,2}\right)\right]\in R^{d_{g}/2},$$

(46) $$g_{t}=\sigma \left(W_{g,3}v_{t,2}+b_{g,3}\right).$$

The implemented sequence is therefore

(47) $$\begin{array}{cc} \mathrm{Linear}(d_{x}+d_{y}+3,d_{g}) & \to \mathrm{GELU}\to \mathrm{Dropout}(p)\\ \; & \to \mathrm{Linear}\left(d_{g},d_{g}/2\right)\to \mathrm{GELU}\to \mathrm{Dropout}\left(p\right)\\ \; & \to \mathrm{Linear}(d_{g}/2,1)\to \mathrm{Sigmoid}. \end{array}$$

The gate performs soft routing rather than hard branch selection. The fused residual is

(48) $$\Delta {\hat{\tilde{y}}}_{t}=g_{t}\Delta {\hat{\tilde{y}}}_{t}^{\;x}+\left(1-g_{t}\right)\Delta {\hat{\tilde{y}}}_{t}^{\;y},$$

and the final standardized estimate is reconstructed as

(49) $${\hat{\tilde{y}}}_{t}={\tilde{y}}_{t-1}+g_{t}\Delta {\hat{\tilde{y}}}_{t}^{\;x}+\left(1-g_{t}\right)\Delta {\hat{\tilde{y}}}_{t}^{\;y}.$$

Because $g_{t}$ is computed independently for each sample, the relative branch contribution can vary throughout the operating sequence. The prediction on the original benchmark scale is obtained using the inverse transformation in Equation (15).

#### 2.3.7. Joint Training Objective

A robust Huber loss is used because the target distribution contains a narrow central region together with infrequent large deviations. For a scalar residual *e* and transition parameter δ, the loss is

(50) $$\ell_{\delta }\left(e\right)=\left\{\begin{array}{cc} \frac{1}{2}e^{2},\; & |e|\leq \delta ,\\ \delta \left(\left|e\right|-\frac{1}{2}\delta \right),\; & |e|>\delta . \end{array}\right.$$

The transition value was fixed at δ=0.05 in the standardized target domain. For a mini-batch of size *B*, the principal prediction loss is

(51) $$L_{\mathrm{pred}}=\frac{1}{B}\sum_{i=1}^{B}\ell_{\delta }\left({\hat{\tilde{y}}}_{i}-{\tilde{y}}_{i}\right).$$

Optimizing only the fused output can permit one expert to become weak while the gate collapses toward the other. To preserve two individually predictive experts, the branch-level estimates in Equations (34) and (40) are supervised through

(52) $$L_{\mathrm{branch}}=\frac{1}{2B}\sum_{i=1}^{B}\left[\ell_{\delta }\left({\hat{\tilde{y}}}_{i}^{\;x}-{\tilde{y}}_{i}\right)+\ell_{\delta }\left({\hat{\tilde{y}}}_{i}^{\;y}-{\tilde{y}}_{i}\right)\right].$$

This auxiliary objective encourages both branches to remain informative before fusion; it does not force their residual corrections to be identical.

A weak gate-balance term can additionally discourage premature batch-level collapse toward a single branch:

(53) $$L_{\mathrm{gate}}={\left(\frac{1}{B}\sum_{i=1}^{B}g_{i}-0.5\right)}^{2}.$$

The regularizer constrains only the average gate value within a mini-batch and does not require every sample to receive equal process and autoregressive weights. The complete objective is

(54) $$L=L_{\mathrm{pred}}+\lambda_{b}L_{\mathrm{branch}}+\lambda_{g}L_{\mathrm{gate}},$$

where $\lambda_{b}$ and $\lambda_{g}$ are selected within the inner hyperparameter-optimization loop. When $\lambda_{g}=0$, routing is governed entirely by the predictive and branch-supervision objectives.

#### 2.3.8. Training-Time Autoregressive Perturbation

To reduce excessive dependence on a perfectly noise-free target history, zero-mean Gaussian perturbation is considered as a training-only regularizer:

(55) $${\tilde{Y}}_{t}^{\;\prime }={\tilde{Y}}_{t}+\epsilon_{t},\;\epsilon_{t}∼N\left(0,\sigma_{\mathrm{AR}}^{2}I\right).$$

When perturbation is active, the residual reference provided to the model is the final perturbed history value,

(56) $${\tilde{y}}_{t-1}^{\;\prime }={\tilde{Y}}_{t}^{\;\prime }\left[-1\right],$$

so that the autoregressive sequence and skip connection remain internally consistent. The process history and regression target remain unperturbed. No noise is applied during validation or outer-test evaluation. The standard deviation $\sigma_{\mathrm{AR}}$ is selected through the inner optimization procedure.

#### 2.3.9. Architecture Summary

Table 2 summarizes the implemented modules and tensor dimensions. GRAG-Net remains compact because each information source is processed by one recurrent layer and the fusion mechanism consists of a small multilayer perceptron.

### 2.4. Comparator Models and Formal Ablations

The revised evaluation was designed to separate three questions: whether target persistence alone is sufficient, whether conventional or sequence-based models can exploit the same causal information, and whether the proposed residual dual-branch gate provides value beyond simpler fusion rules. All learnable models were fitted using only information available within the corresponding outer-training period.

Two parameter-free references were included. Persistence uses ${\hat{y}}_{t}=y_{t-1}$, while linear extrapolation extends the most recent target increment, ${\hat{y}}_{t}=2y_{t-1}-y_{t-2}$. Four classical regressors—SVR [23], Random Forest [24], Extra Trees [25], and XGBoost [26]—were evaluated in both absolute-target ARX form and residual form. The residual variants predict $\Delta y_{t}=y_{t}-y_{t-1}$ and reconstruct the target by adding the predicted increment to $y_{t-1}$; this control is important because it separates the effect of residual learning from the recurrent architecture.

The sequence-model controls comprised a strict process-only GRU, an AR–GRU using only causal target history, a joint GRU receiving combined process and target history information, LSTM–ARX, and TCN–ARX. The process-only GRU receives neither target history samples nor the $y_{t-1}$ residual anchor. Two fusion controls use the same dual-branch information as GRAG-Net: equal fusion fixes the branch weight at 0.5, whereas the global-gate model learns one scalar fusion weight shared by all samples. These controls distinguish the value of sample-dependent routing from the mere availability of two branches.

Formal ablations were independently trained for the full GRAG-Net, removal of the residual reconstruction, equal fusion, global gating, removal of branch supervision ($\lambda_{b}=0$), removal of gate-balance regularization ($\lambda_{g}=0$), removal of branch disagreement $d_{t}$, and removal of the process-change statistic $s_{t}$. AR-history perturbation is not interpreted as an active component of the selected final model because hyperparameter selection returned $\sigma_{\mathrm{AR}}=0$ in all three outer folds.

### 2.5. Chronological Model Development and Reproducibility

#### 2.5.1. Nested Chronological Design

Random partitioning was avoided because neighboring SRU samples are strongly autocorrelated and time-ordered evaluation is required to avoid optimistic performance estimates in autoregressive settings [27]. Three expanding outer-training periods were therefore followed by non-overlapping future evaluation periods, as summarized in Table 3. The outer-test targets were excluded from model fitting, preprocessing, hyperparameter selection, and epoch selection. During test-time rolling one-step evaluation, however, an SO_2_ observation becomes admissible historical information after its time has passed; thus a prediction at *t* may use measured target history only through t−1. Predicted targets are never recursively fed back.

**Figure 5:**
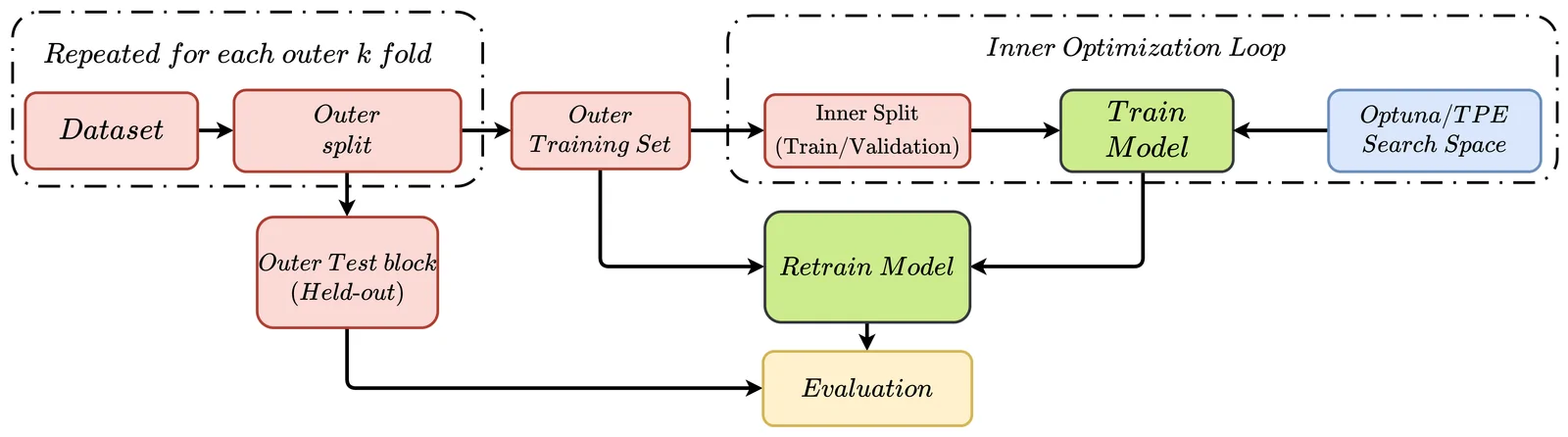
Leakage-aware nested chronological model development. Hyperparameters, preprocessing parameters, and epoch-selection decisions are determined using only the outer-training period. The subsequent future block is held out from model development and is evaluated under causal rolling one-step inference, where measured SO_2_ history is available only through the preceding time instant.

Figure 5 summarizes the nested workflow. Within each outer-training period, the earliest 80% of observations formed the inner-training subset and the latest 20% formed the inner-validation subset. Scaling parameters were estimated only from the active training subset. Optuna [28] with a seeded tree-structured Parzen estimator (2026) was used for configuration selection. Final neural models were evaluated with seeds {42,52,62,72,82} after all model-selection decisions had been fixed.

#### 2.5.2. Hyperparameter Search Spaces and Selected Configuration

To make the revised comparison reproducible, the search domains used for the reviewer-requested models are reported explicitly in Table 4. Search was performed independently inside each outer-training period using the chronological inner split described above; the outer-test block was never used to choose a configuration. Classical models used 18 Optuna trials per fold and neural models used 24 trials per fold. The final reviewer run reused the winning configurations saved from this model development stage, without test-set retuning.

The full GRAG-Net configuration selected independently in each outer fold was identical across all three folds, as summarized in Table 5. This agreement emerged from independent inner-fold selection; the configuration was not imposed globally. In particular, $\sigma_{\mathrm{AR}}=0$ was selected in every fold, so autoregressive history perturbation is not treated as an active component of the final model. Exact fold-specific winning configurations for every comparator and formal ablation, together with the corresponding Optuna trial histories, were retained in the reproducibility outputs. These records are available with the revision materials and permit reconstruction of every reported model-selection decision.

After hyperparameter selection, the final training length for each neural model, outer fold, and seed was determined without using the outer-test block. Specifically, the earliest 85% of the corresponding outer-training period was used for fitting and the latest 15% for epoch selection, with a maximum of 150 epochs and early-stopping patience of 15. The selected epoch was then used to retrain the model from a new initialization on the complete outer-training period before evaluation. Thus, the temporary epoch-selection subset was returned to the final training set and no test observation was used to determine training duration.

The final revision was executed under Windows 11 using Python 3.13.9, NumPy 2.3.5, pandas 2.3.3, PyTorch 2.11.0 with CUDA 12.6, scikit-learn 1.7.2, XGBoost 3.2.0, and SHAP 0.51.0 on an NVIDIA T1000 8 GB GPU. The exact chronological splits, selected configurations, fitted preprocessing parameters, epoch-selection histories, model checkpoints, fold–seed predictions, metrics, and diagnostic outputs were retained as a reproducibility audit trail.

### 2.6. Evaluation, Statistical Analysis, and Diagnostics

All predictions were inverse transformed before evaluation, and SO_2_ errors are reported in mole%. The primary predictive measures are MAE, RMSE, and $R^{2}$; sMAPE, median absolute error, explained variance, mean bias deviation, and Pearson correlation are retained as secondary diagnostics. Because the five random seeds within one chronological fold share the same test period, the three outer folds are treated as the primary independent evaluation periods; seed dispersion describes optimization variability rather than five additional independent datasets.

For fold *k*, the main error measures are

(57) $${\mathrm{MAE}}^{\left(k\right)}=\frac{1}{n_{k}}\sum_{i=1}^{n_{k}}\left|y_{i}-{\hat{y}}_{i}\right|,\;{\mathrm{RMSE}}^{\left(k\right)}=\sqrt{\frac{1}{n_{k}}\sum_{i=1}^{n_{k}}{\left(y_{i}-{\hat{y}}_{i}\right)}^{2}}.$$

The pooled seed-ensemble analysis averages the five predictions for each unique out-of-fold sample before computing metrics over the 4537 non-overlapping test observations. It is reported as a complementary aggregate, not as a replacement for fold-wise evidence.

Taylor diagrams are reported separately for the three outer folds. Each model is located by its Pearson correlation and predicted standard deviation, while centered RMSE contours summarize joint discrepancies in phase and variability. The ideal point has correlation one, predicted standard deviation equal to the observed standard deviation, and zero centered RMSE [29].

To quantify uncertainty while respecting serial dependence, paired moving-block bootstrap analysis was applied to sample-wise loss differences

(58) $$\Delta_{t}=\ell \left(y_{t},{\hat{y}}_{\mathrm{GRAG},t}\right)-\ell \left(y_{t},{\hat{y}}_{B,t}\right),$$

using absolute and squared error losses. A block length of 30 samples is the primary analysis, with 15 and 60 samples retained as sensitivity checks. Negative Δ favors GRAG-Net. Confidence intervals are computed within each chronological test period rather than pooling folds as if they were exchangeable independent replicates.

The diagnostic analysis was deliberately separated into inference-available and retrospective quantities. Gate behavior was summarized by Spearman association between the process-branch weight $g_{t}$ and three quantities available at inference: branch disagreement $d_{t}$, process change $s_{t}$, and the latest measured target $y_{t-1}$. Any association with realized branch advantage is treated only as retrospective interpretation because it depends on $y_{t}$.

Temporal alignment was examined using training-only lagged Pearson cross-correlations between each process variable and SO_2_. For each fold, the reported lag is the training-period lag that maximizes |r|; these correlations are descriptive and are not interpreted as causal delay estimates. Error reliability was assessed using the empirical CDF and the 50th, 75th, 90th, 95th, and 99th percentiles of absolute error. A retrospective target-change analysis partitions test samples by $|y_{t}-y_{t-1}|$: the lower 50% form the stable regime, the 50th–90th percentiles form the changing regime, and the upper 10% form the large-change regime. This analysis tests whether conclusions depend primarily on quasi-steady observations.

SHAP was computed for two predefined seeds (42 and 52) in each of the three folds. Global importance is based on mean absolute SHAP value aggregated across these six fitted artifacts. Explanation stability is quantified by pairwise Spearman correlation of feature ranks and top-20 Jaccard overlap. SHAP values describe the fitted model and should not be interpreted as causal process effects [30,31].

The public benchmark and the originating SRU literature report SO_2_ concentration in mole%. The released benchmark does not document the make, calibration uncertainty, accuracy specification, or regulatory tolerance of the plant analyzer used to obtain the reference SO_2_ measurements. Because these instrument-specific quantities cannot be recovered reliably from the available data, they are not inferred or approximated here. Accordingly, the study reports prediction errors in the available physical concentration unit but does not compare them with an unspecified analyzer-accuracy or compliance threshold.

## 3. Results and Discussion

The revised experiments assess GRAG-Net under the causal rolling one-step setting defined in Section 2.2. The three non-overlapping chronological test periods are treated as the primary evaluation periods. Five final seeds quantify optimization variability within each fold, while pooled seed-ensemble predictions are reported only as a complementary sample-level summary over the 4537 unique out-of-fold observations.

### 3.1. Comparative Predictive Performance

Table 6 reports descriptive mean ± standard deviation over the 15 fold–seed evaluations for each model. These values summarize both chronological and initialization variability; they are not interpreted as 15 independent datasets because the five seeds within a fold share the same test observations. GRAG-Net achieved the strongest primary performance, with MAE 0.00315±0.00044 mole%, RMSE 0.00790±0.00064 mole%, and $R^{2}=0.97753\pm 0.00331$. It also obtained the highest Pearson correlation (0.98898±0.00156) and the lowest sMAPE (2.258±0.462%) among the learned full-model comparators.

The strongest sequence comparator was LSTM–ARX in RMSE and $R^{2}$, whereas linear extrapolation produced the second-lowest MAE. Nevertheless, neither matched GRAG-Net simultaneously in absolute error, squared-error sensitivity, variance explanation, and correlation. The dedicated AR–GRU was also substantially weaker, with MAE 0.00453±0.00052 and RMSE 0.00979±0.00086 mole%. Thus, the gain of GRAG-Net cannot be attributed merely to access to recent target history; the exogenous branch and adaptive residual fusion provide additional predictive information beyond a strong autoregressive recurrent model.

MSE and explained variance were also computed and retained in the reproducibility outputs; they are omitted from the main table because they are largely redundant with RMSE and $R^{2}$. The residual classical controls additionally show that residualization alone is insufficient: residual SVR is the strongest classical residual model, but its MAE and RMSE remain above those of GRAG-Net. Likewise, equal fusion and global gating improve substantially over the strict process-only model but remain behind the dynamic gate, supporting the value of sample-dependent fusion rather than simply adding a second branch.

The pooled seed-ensemble analysis leads to the same conclusion. GRAG-Net attained MAE 0.00291 mole%, RMSE 0.00766 mole%, and $R^{2}=0.97937$. Relative to persistence, these correspond to MAE and RMSE reductions of 55.2% and 44.8%; relative to AR–GRU, the reductions are 32.5% and 18.0%. Because pooling averages the five predictions for each unique sample, these values are complementary ensemble statistics and are not substituted for the fold-level evidence below.

### 3.2. Chronological Robustness and Temporal Tracking

Table 7 reports fold-wise mean performance over the five final seeds for the principal sequence and fusion models. GRAG-Net has the lowest MAE and RMSE in all three chronological periods. Its MAE ranges from 0.00285 to 0.00372 mole%, while $R^{2}$ remains between 0.97344 and 0.97980. The third fold is the most difficult in absolute-error terms, yet the proposed model preserves high explained variance and maintains a clear margin over AR–GRU, LSTM–ARX, TCN–ARX, equal fusion, and global gating.

**Figure 6:**
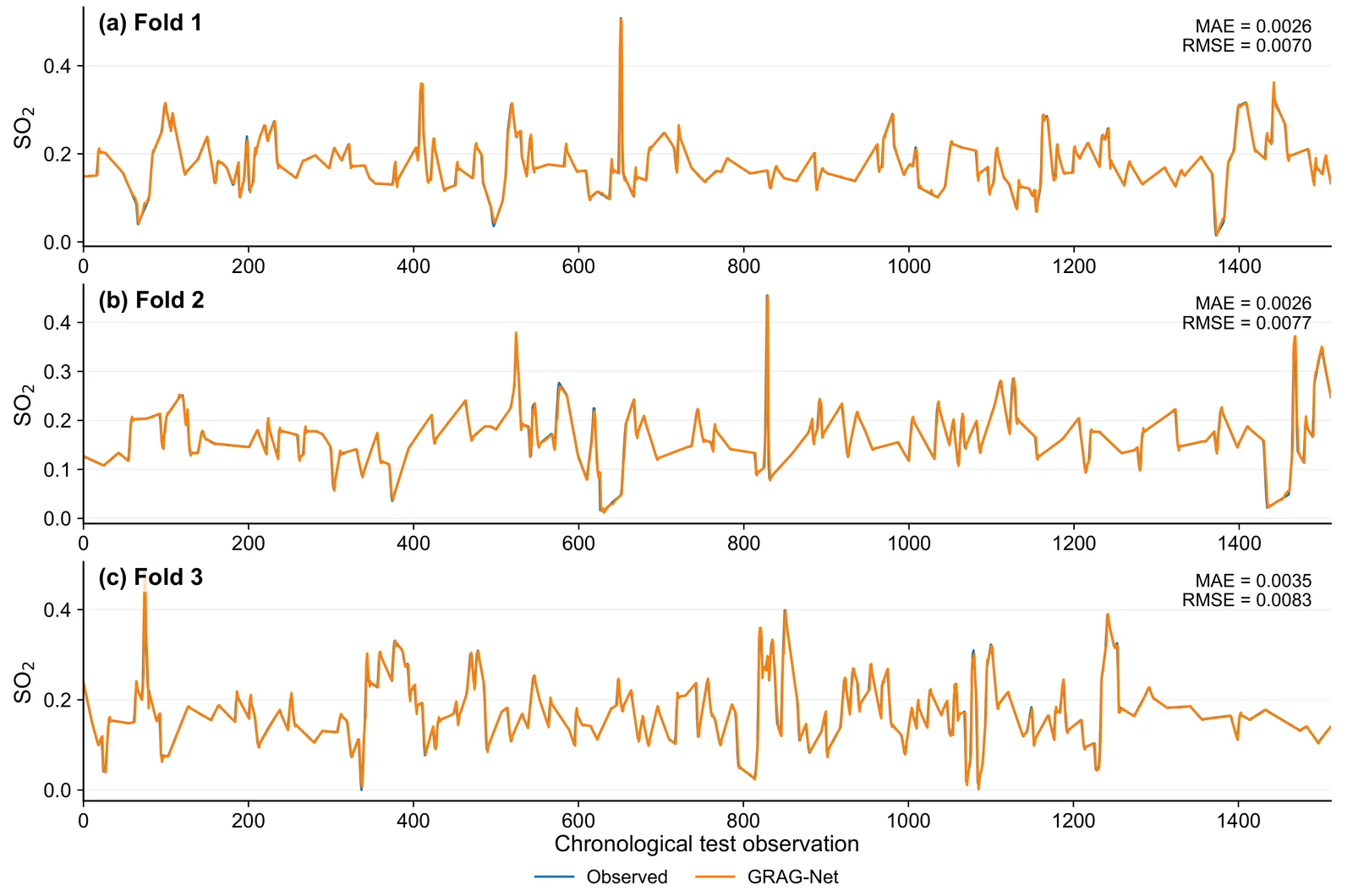
Observed and GRAG-Net-predicted SO_2_ concentration over the three chronological outer-test periods. Predictions are seed-ensemble means; fold-specific MAE and RMSE are reported in each panel.

Figure 6 shows the seed-ensemble GRAG-Net trajectories. The ensemble MAE values are 0.002635, 0.002565, and 0.003534 mole% for Folds 1–3, respectively, with corresponding RMSE values of approximately 0.00695, 0.00769, and 0.00827 mole%. The predicted trajectories closely follow the observed concentration through both steady and rapidly varying intervals, with the largest deviations concentrated around abrupt excursions.

### 3.3. Taylor-Diagram Assessment

**Figure 7:**
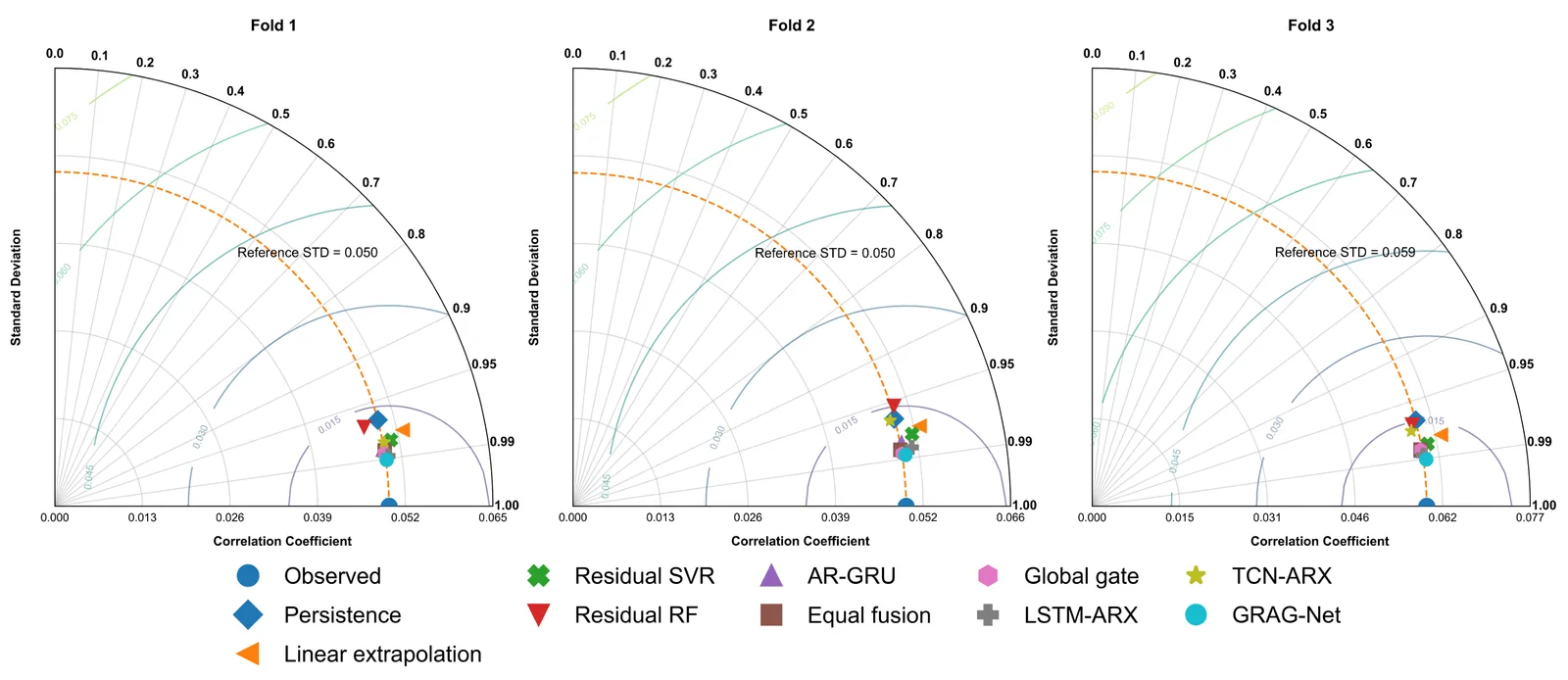
Taylor diagrams for the three chronological test folds. Radial distance denotes predicted standard deviation, angular position denotes Pearson correlation, and contours denote centered RMSE. The observation is the reference point.

The fold-specific Taylor diagrams in Figure 7 provide a joint view of correlation, variance reproduction, and centered error. GRAG-Net attains correlations of 0.99035, 0.98829, and 0.99030 in Folds 1–3 and the lowest centered RMSE in every fold: 0.00695, 0.00769, and 0.00826 mole%, respectively. Its predicted standard deviations (0.05010, 0.05040, and 0.05948) closely match the corresponding observed values (0.04997, 0.04992, and 0.05898). These results indicate that the proposed model not only reduces pointwise error but also reproduces the amplitude and temporal variability of the observed SO_2_ sequence more faithfully than the evaluated alternatives.

### 3.4. Formal Ablation of the Proposed Architecture

**Figure 8:**
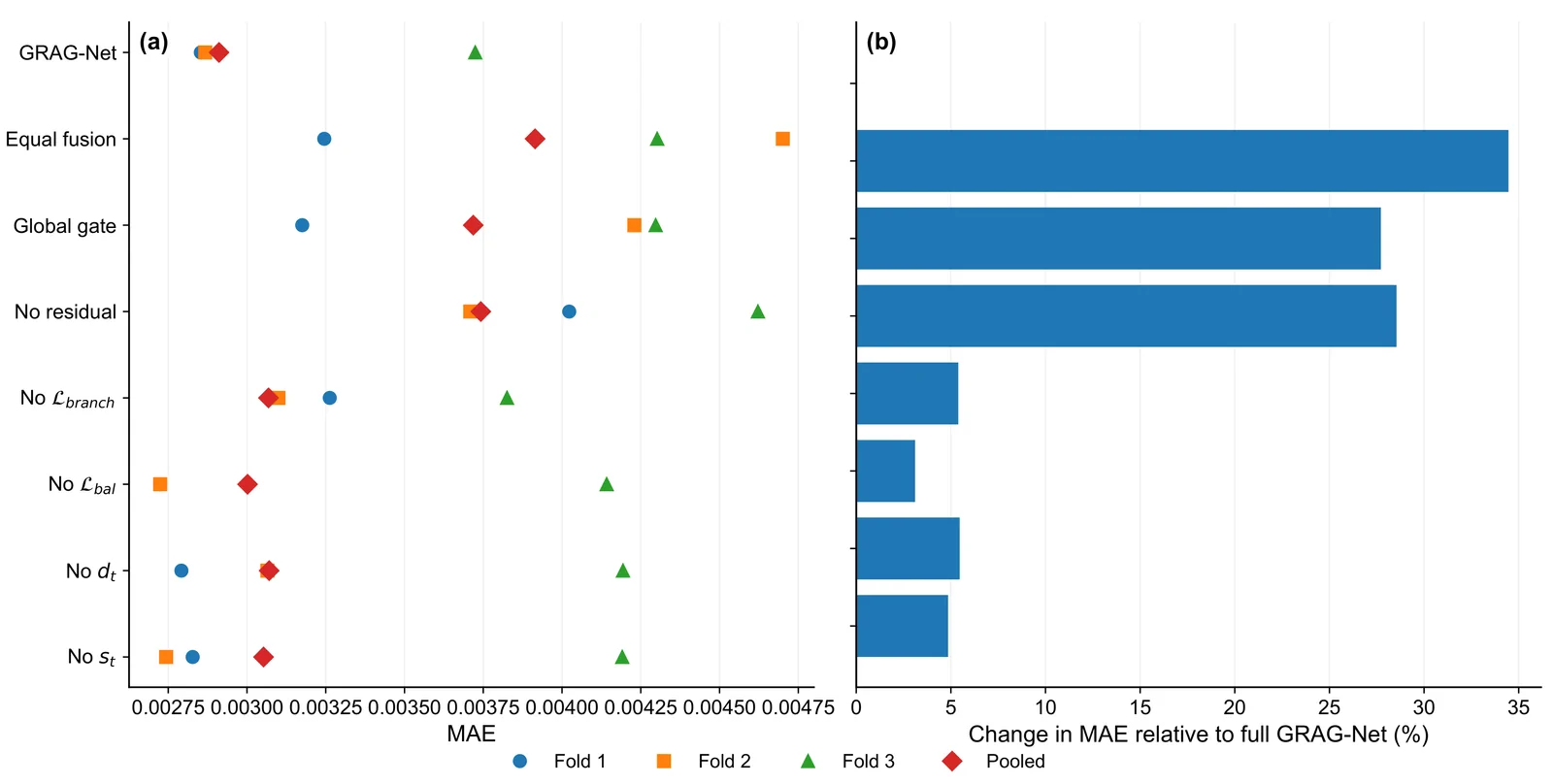
Formal independently trained ablations. (**a**) Fold-wise and pooled MAE. (**b**) Pooled percentage change in MAE relative to the full GRAG-Net. Positive values denote degradation relative to the full model.

Figure 8 summarizes independently trained and re-optimized ablations. Relative to the full model’s pooled MAE of 0.00291 mole%, fixed equal fusion increases MAE by 34.5%, a global gate by 27.7%, and removal of residual reconstruction by 28.5%. Removing branch supervision, disagreement, process-change context, or gate-balance regularization produces smaller pooled degradations of approximately 3–5.5%. The dominant performance losses therefore occur when the residual formulation or sample-dependent fusion mechanism is simplified, directly supporting the two central design choices of GRAG-Net.

The smaller components show more modest and fold-dependent effects and should be interpreted as regularizing or contextual refinements rather than as uniformly dominant mechanisms. In particular, the “no AR noise” row is a re-optimized control rather than evidence that perturbation itself is beneficial: the selected full-model configuration has $\sigma_{\mathrm{AR}}=0$, so autoregressive perturbation is inactive in the final GRAG-Net. This distinction prevents attributing a causal benefit to a component that was not used by the selected model.

### 3.5. Paired Moving-Block Bootstrap Evidence

**Figure 9:**
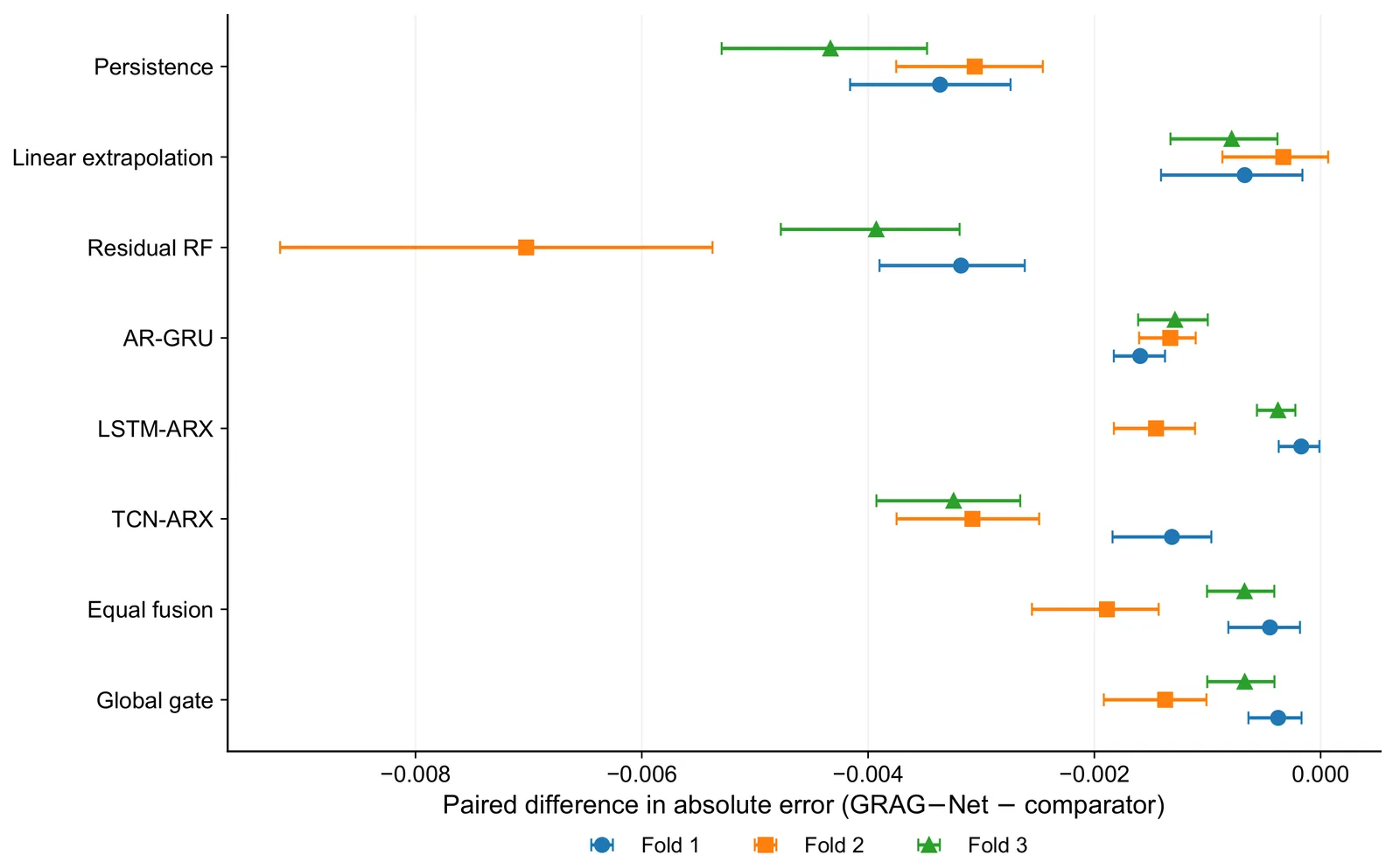
Paired moving-block bootstrap differences in absolute error for the primary block length of 30 samples. Each point is the mean of $|e_{\mathrm{GRAG}}\left|\;-\;\right|e_{B}|$ and the horizontal interval is the 95% bootstrap confidence interval. Negative values favor GRAG-Net.

The paired moving-block bootstrap provides dependence-aware uncertainty for sample-wise error differences within each chronological test period. Figure 9 reports the primary 30-sample block analysis for absolute error, where negative values of $|e_{\mathrm{GRAG}}\left|\;-\;\right|e_{B}|$ favor GRAG-Net. Against AR–GRU, the mean differences are −0.001595, −0.001329, and −0.001288 mole% in Folds 1–3, and all three 95% confidence intervals lie entirely below zero. GRAG-Net is likewise favored in all folds against LSTM–ARX, TCN–ARX, equal fusion, global gating, persistence, residual RF, and residual SVR. Linear extrapolation is inferior in Folds 1 and 3, while its Fold 2 interval slightly overlaps zero.

This analysis strengthens the descriptive metric comparison because it preserves temporal pairing and serial dependence rather than treating individual time samples as independent. The conclusion is also insensitive to the alternative block lengths of 15 and 60 samples retained in the sensitivity analysis: the principal comparisons remain directionally consistent. Thus, the advantage of GRAG-Net over the strongest recurrent controls is not explained solely by averaging across seeds or by a small number of isolated test points.

### 3.6. Gate Behavior and Training-Only Temporal Lags

**Figure 10:**
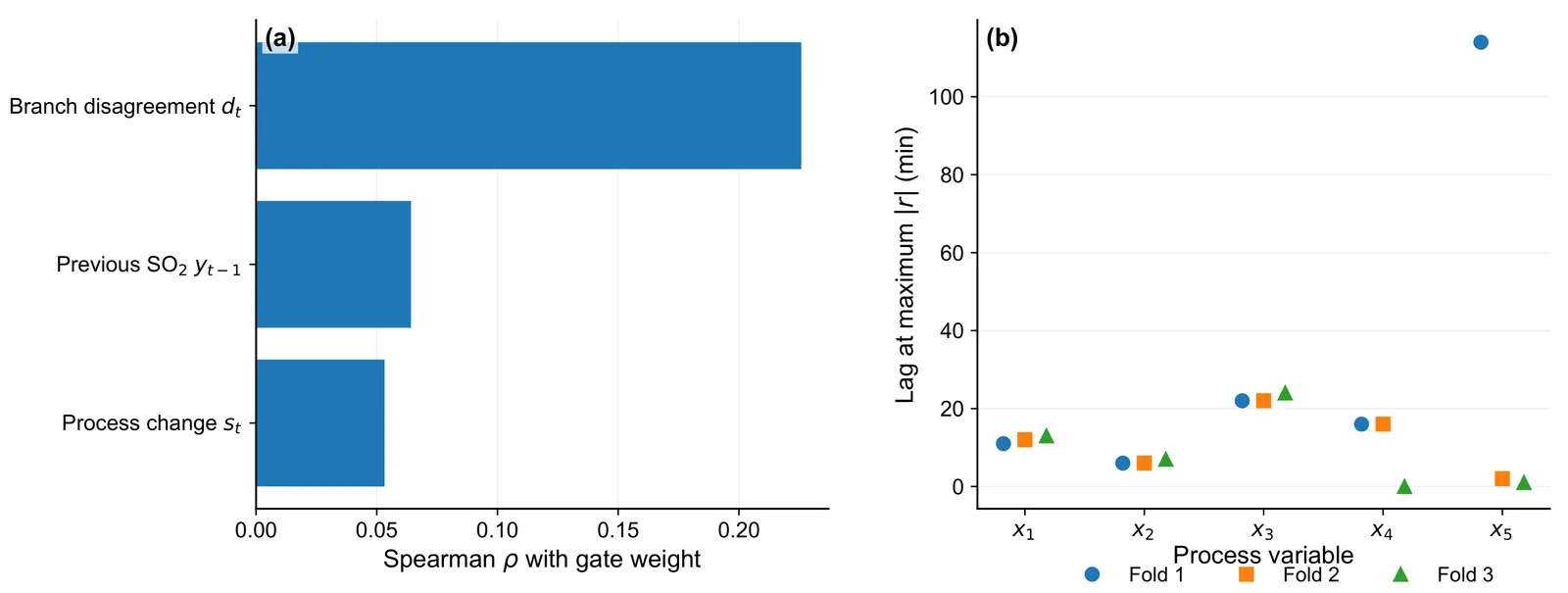
Inference-aware gate and temporal-lag diagnostics. (**a**) Pooled Spearman association between gate weight and causal gate-context variables. (**b**) Training-only lag at maximum absolute process–SO_2_ correlation in each fold.

The inference-aware gate analysis in Figure 10a shows that the process-branch weight is most strongly associated with branch disagreement (ρ=0.226), whereas associations with the process-change statistic (ρ=0.053) and the latest SO_2_ level (ρ=0.064) are weak. The gate should therefore be interpreted as a learned routing mechanism that reacts primarily to disagreement between the proposed process and autoregressive corrections, with weaker modulation by current process variation and target level. The retrospective association with realized process-branch advantage is only ρ=0.122 and is not treated as causal evidence because it depends on the subsequently observed target.

Training-only lag analysis in Figure 10b identifies the clearest delayed linear relationships for $x_{1}$, $x_{2}$, and $x_{3}$, whose maximum absolute correlations generally occur about 6–24 min before the SO_2_ response. The corresponding |r| values are modest (approximately 0.13–0.21), indicating that no single process variable provides a dominant linear predictor. The Fold 1 maximum for $x_{5}$ occurs at 114 min but has only |r|≈0.049; this weak boundary-like maximum is therefore not interpreted as a physically precise delay. The lag analysis supports the use of temporal process histories while reinforcing the need for a nonlinear multivariate representation.

### 3.7. SHAP Interpretation and Stability

**Figure 11:**
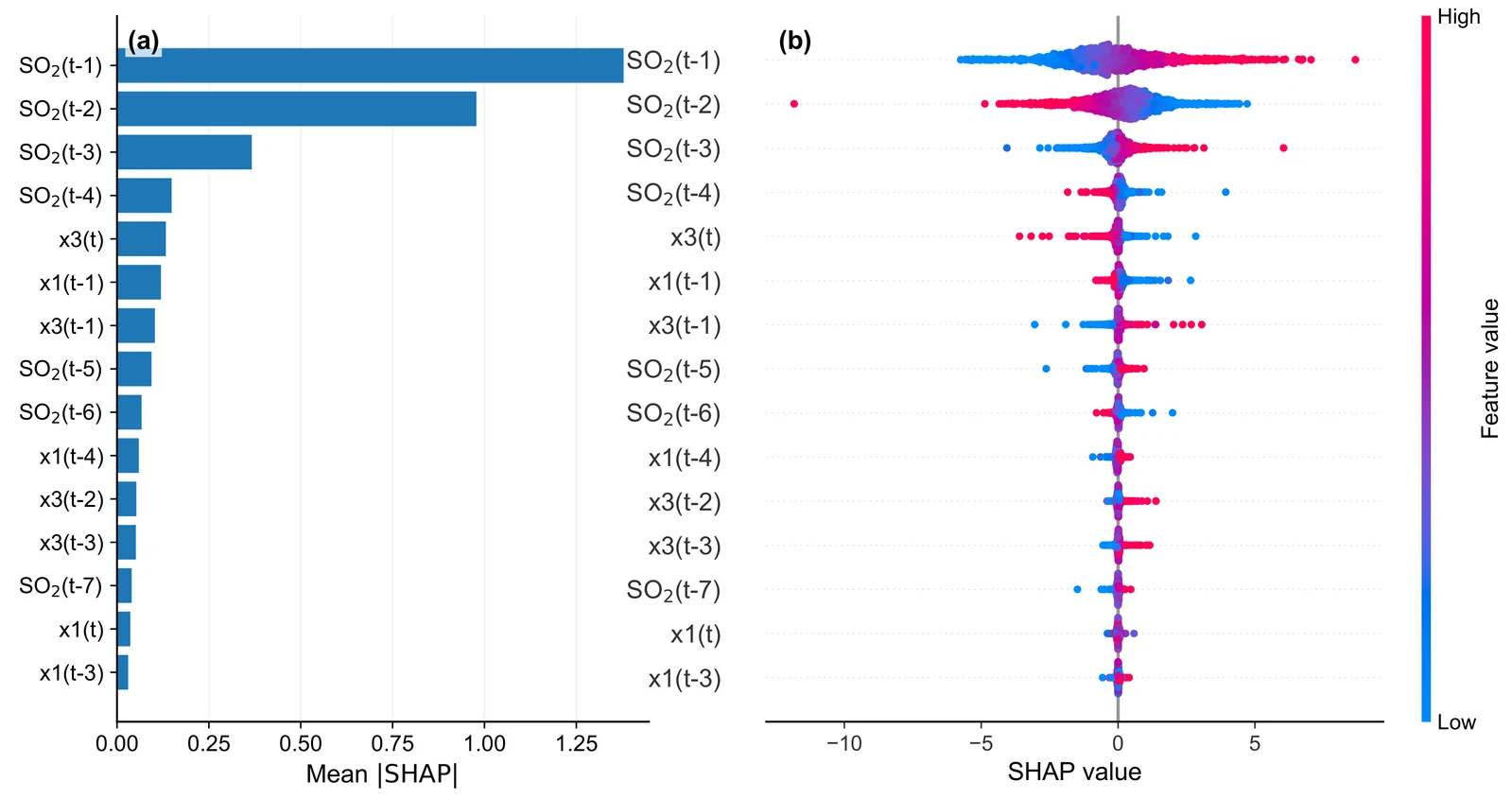
Aggregated SHAP interpretation of GRAG-Net. (**a**) Mean absolute SHAP importance for the 15 leading lagged features. (**b**) SHAP beeswarm showing the direction and magnitude of sample-level contributions.

The aggregated SHAP analysis in Figure 11 confirms that recent SO_2_ history provides the dominant persistence information. The four highest-ranked features are $y_{t-1}$ through $y_{t-4}$, with mean absolute SHAP values of 1.379, 0.978, 0.367, and 0.149, respectively. The strongest exogenous contributions are current $x_{3}$, $x_{1}\left(t-1\right)$, and $x_{3}\left(t-1\right)$. This ordering is physically consistent with the architecture: the autoregressive branch supplies the dominant local baseline, while airflow histories provide complementary information for correcting the estimate.

The beeswarm representation further indicates nonlinear and sample-dependent effects rather than a single fixed coefficient for each process lag. These attributions describe the fitted predictor and should not be interpreted as causal plant effects, particularly because neighboring lags and airflow channels are correlated. Importantly, the revision no longer relies on one fitted artifact. Across the six predefined fold–seed artifacts (three folds and seeds 42 and 52), the mean pairwise rank Spearman correlation is 0.933 (range 0.906–0.984), and the mean top-20 Jaccard overlap is 0.653 (range 0.538–0.818). The dominant feature ordering is therefore stable across chronological periods and random initialization, while lower-ranked features show the expected greater variability.

**Figure 12:**
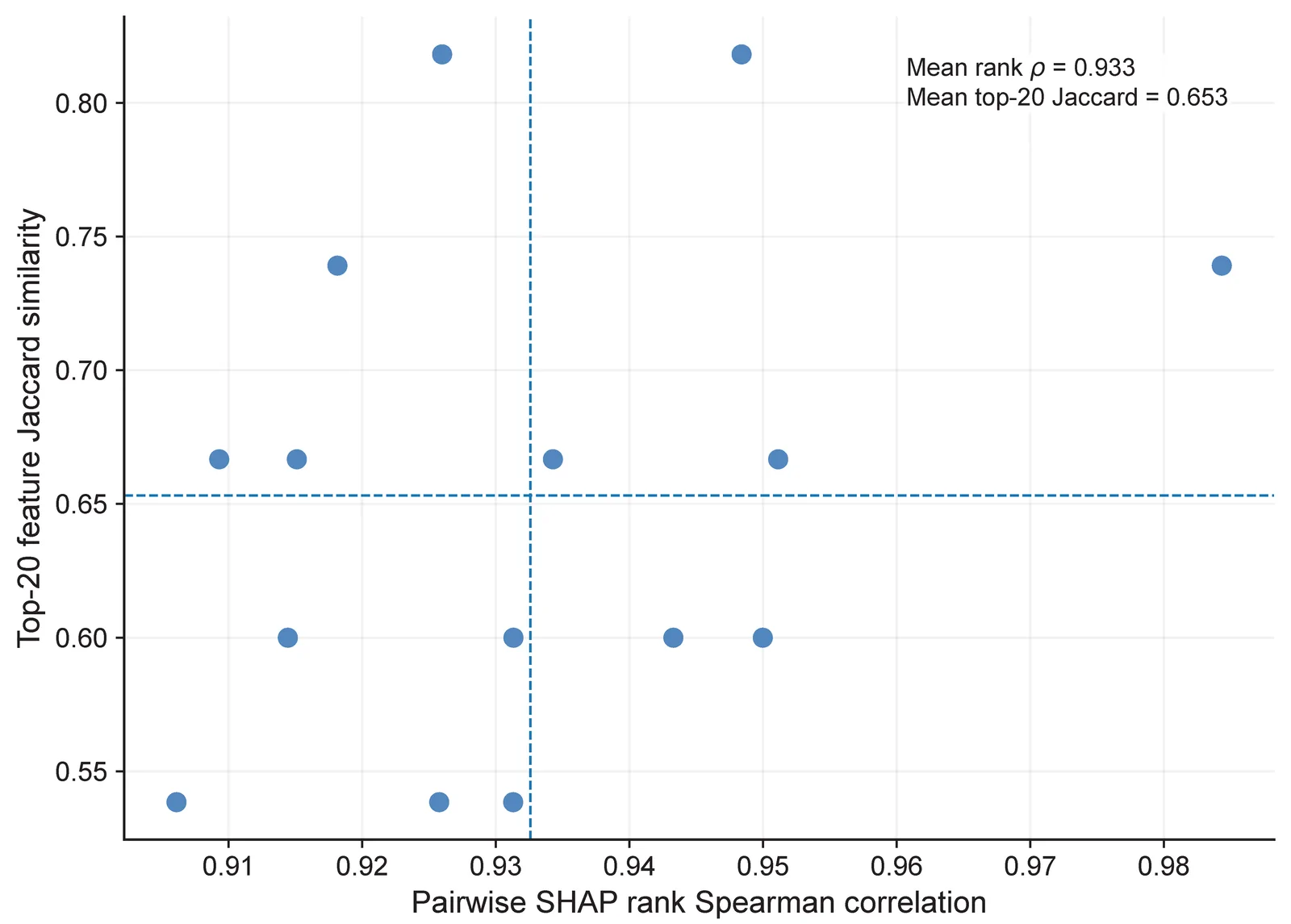
Cross-fold and cross-seed stability of SHAP rankings. Each point compares two fitted artifacts using full-rank Spearman correlation and top-20 Jaccard overlap; dashed lines denote the corresponding means.

Figure 12 summarizes the consistency of SHAP feature rankings across folds and seeds using Spearman correlation and top-20 Jaccard overlap.

### 3.8. Error Reliability and Target-Change Regimes

**Figure 13:**
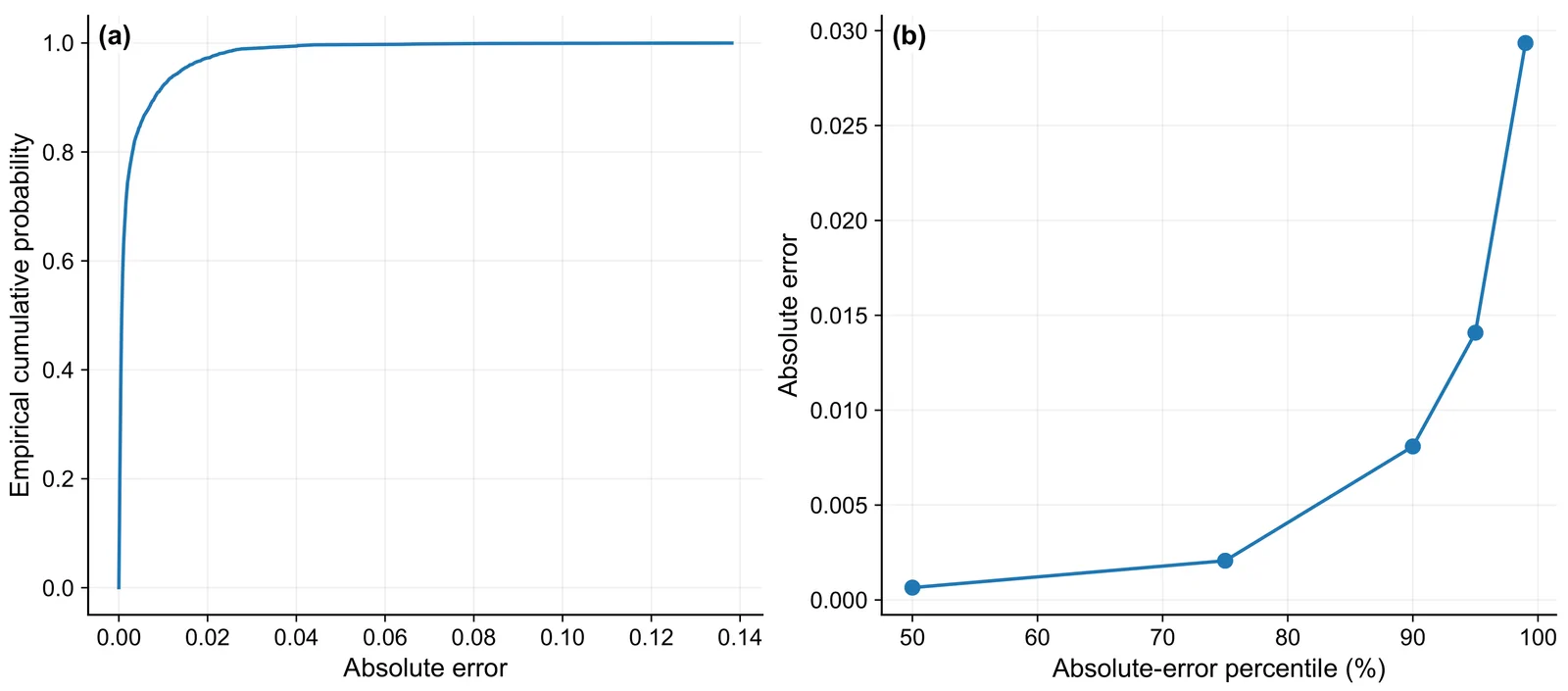
GRAG-Net error reliability. (**a**) Empirical CDF of absolute error. (**b**) Selected absolute-error percentiles, highlighting the concentration of typical errors and the upper tail.

The error-reliability analysis in Figure 13 shows that GRAG-Net improves both the center and the upper tail of the error distribution relative to AR–GRU. Its median absolute error is $6.51\times 10^{-4}$ mole%, compared with $1.74\times 10^{-3}$ mole% for AR–GRU. At the 90th, 95th, and 99th percentiles, GRAG-Net errors are 0.00809, 0.01409, and 0.02935 mole%, whereas AR–GRU reaches 0.01080, 0.01866, and 0.03334 mole%, respectively. The performance gain is therefore not confined to a few extreme observations; it extends from typical errors through the upper tail.

The target-change analysis gives a complementary view. GRAG-Net attains MAE/RMSE of 0.00095/0.00210 mole% in the stable regime, 0.00243/0.00444 mole% in the changing regime, and 0.01465/0.02202 mole% during large changes. The corresponding AR–GRU values are 0.00131/0.00225, 0.00412/0.00553, and 0.02014/0.02690 mole%. GRAG-Net therefore outperforms the dedicated autoregressive recurrent model in all three regimes, with the largest absolute margin occurring during rapid target changes. This finding supports the intended role of the process branch: exogenous histories provide corrective information that is useful not only during transitions but also during routine one-step estimation.

Overall, the revised evidence is internally consistent across descriptive metrics, chronological folds, Taylor statistics, block-bootstrap intervals, formal ablations, and error-regime analysis. GRAG-Net does not obtain its advantage from target persistence alone: AR–GRU, linear extrapolation, and fixed/global fusion controls all remain weaker. Instead, the combination of residual reconstruction, separate process and autoregressive representations, and sample-dependent routing yields the most accurate and stable one-step predictions among the evaluated models. The gate, lag, and SHAP analyses further support a cautious mechanistic interpretation in which recent SO_2_ history provides the dominant baseline and process history supplies complementary corrections when the two predictive pathways disagree.

## 4. Conclusions and Future Work

This study developed GRAG-Net for causal rolling one-step SO_2_ soft sensing in an industrial sulfur recovery unit. The architecture separates exogenous process dynamics from autoregressive target persistence, predicts residual corrections relative to the latest measured SO_2_ value, and combines the two branches through a sample-dependent gate. The major revision strengthens the evidence for this design through three chronological future periods, five final seeds, stronger sequence and residual baselines, independently trained ablations, and dependence-aware moving-block bootstrap analysis.

Across the 15 descriptive fold–seed evaluations, GRAG-Net achieved MAE 0.00315±0.00044 mole%, RMSE 0.00790±0.00064 mole%, and $R^{2}=0.97753\pm 0.00331$, providing the strongest overall primary-metric performance among the evaluated full models. On pooled seed-ensemble predictions, MAE and RMSE were 0.00291 and 0.00766 mole%, representing reductions of 55.2% and 44.8% relative to persistence and 32.5% and 18.0% relative to AR–GRU. GRAG-Net also produced the lowest centered RMSE in all three Taylor folds, lower absolute-error quantiles from the median through the 99th percentile than AR–GRU, and lower MAE and RMSE in stable, changing, and large-change regimes. Paired moving-block bootstrap intervals favor GRAG-Net over AR–GRU in all three folds for absolute error, further supporting that the improvement is consistent within the chronological test periods rather than being driven by pooled averaging alone.

The formal ablations identify residual reconstruction and sample-dependent fusion as the principal architectural contributors: replacing dynamic routing with equal fusion, replacing it with a global gate, or removing residual reconstruction increases pooled MAE by approximately 34.5%, 27.7%, and 28.5%, respectively. Auxiliary losses and individual gate-context variables have smaller and more fold-dependent effects. Inference-aware gate analysis shows that routing is associated most strongly with branch disagreement, while training-only lag analysis identifies modest delayed relationships for the principal airflow channels. Aggregated SHAP analysis confirms that recent SO_2_ lags dominate prediction and that process variables provide complementary exogenous information; the feature ranking remains stable across folds and seeds (mean rank ρ=0.933).

The conclusions remain limited to one publicly available SRU benchmark and to a rolling one-step setting in which measured SO_2_ history is available through t−1. The study does not evaluate sustained analyzer outages, recursive multi-step prediction, cross-refinery transfer, or closed-loop deployment. Because the benchmark does not document analyzer accuracy or regulatory tolerance, the reported model errors are not interpreted as evidence of compliance with a particular hardware specification. Future work should prioritize external validation on additional SRUs, recursive outage-oriented evaluation, uncertainty calibration, drift-aware adaptation, and prospective deployment studies under documented analyzer specifications.

## Figures and Tables

**Table 1:** Descriptive statistics of the industrial SRU benchmark (N= 10,081).

Variable	Mean	Std.	Minimum	$Q_{1}$	Median	$Q_{3}$	Maximum
MEA airflow ($x_{1}$)	0.5623	0.2054	0.0000	0.4130	0.5502	0.6977	1.0000
SWS airflow ($x_{2}$)	0.5623	0.2277	0.0000	0.3567	0.5857	0.7678	1.0000
Airflow in the MEA zone ($x_{3}$)	0.4461	0.1519	0.0000	0.3420	0.4494	0.5360	1.0000
Airflow in the SWS zone ($x_{4}$)	0.5940	0.2420	0.0000	0.4100	0.7225	0.7395	1.0000
Secondary airflow ($x_{5}$)	0.6011	0.2029	0.0000	0.5091	0.6953	0.7039	1.0000
SO_2_ concentration (mole%)	0.1706	0.0569	0.0000	0.1435	0.1665	0.1923	1.0000

**Table 2:** Implemented GRAG-Net modules, layer configurations, and output dimensions.

Module	Layer Sequence or Operation	Output Dimension
Process encoder	Single-layer GRU with input size 5 and hidden size $d_{x}$	$h_{t}^{x}\in R^{d_{x}}$
Process projection	Linear$\left(d_{x},d_{x}\right)$–GELU–Dropout(p)–LayerNorm	$z_{t}^{x}\in R^{d_{x}}$
Process correction head	Linear$\left(d_{x},d_{x}/2\right)$–GELU–Dropout(p)–Linear$\left(d_{x}/2,1\right)$	$\Delta {\hat{\tilde{y}}}_{t}^{\;x}\in R$
Autoregressive encoder	Single-layer GRU with input size 1 and hidden size $d_{y}$	$h_{t}^{y}\in R^{d_{y}}$
Autoregressive projection	Linear$\left(d_{y},d_{y}\right)$–GELU–Dropout(p)–LayerNorm	$z_{t}^{y}\in R^{d_{y}}$
Autoregressive correction head	Linear$\left(d_{y},d_{y}/2\right)$–GELU–Dropout(p)–Linear$\left(d_{y}/2,1\right)$	$\Delta {\hat{\tilde{y}}}_{t}^{\;y}\in R$
Gate context	$\begin{array}{c} c_{t}={\left[{\left(z_{t}^{x}\right)}^{⊤},{\left(z_{t}^{y}\right)}^{⊤},{\tilde{y}}_{t-1},d_{t},s_{t}\right]}^{⊤} \end{array}$	$c_{t}\in R^{d_{x}+d_{y}+3}$
Reliability gate	$\begin{array}{c} \mathrm{Linear}\left(d_{x}+d_{y}+3,d_{g}\right)–\mathrm{GELU}–\mathrm{Dropout}\left(p\right)\\ –\mathrm{Linear}\left(d_{g},d_{g}/2\right)–\mathrm{GELU}–\mathrm{Dropout}\left(p\right)\\ –\mathrm{Linear}(d_{g}/2,1)–\mathrm{Sigmoid} \end{array}$	$g_{t}\in \left[0,1\right]$
Residual reconstruction	$\begin{array}{c} {\hat{\tilde{y}}}_{t}={\tilde{y}}_{t-1}+g_{t}\Delta {\hat{\tilde{y}}}_{t}^{\;x}+\left(1-g_{t}\right)\Delta {\hat{\tilde{y}}}_{t}^{\;y} \end{array}$	${\hat{\tilde{y}}}_{t}\in R$

**Table 3:** Chronological outer folds used for final evaluation. Intervals are half-open index ranges.

Fold	Training Interval	Training Size	Test Interval	Test Size
1	[0,5544)	5544	[5544,7056)	1512
2	[0,7056)	7056	[7056,8568)	1512
3	[0,8568)	8568	[8568, 10,081)	1513

**Table 4:** Hyperparameter search spaces used in the nested model development stage. Parameters not listed were fixed by the implementation. Structural ablations used the same numerical search domain while forcing the ablated component to its prescribed setting.

Model Family	Hyperparameter(s)	Search Domain
All learnable models	$L_{x}$, $L_{y}$	{16,32,64}; {4,8,16,32}
SVR–ARX/residual SVR	*C*, ε, γ	$\left[10^{-1},10^{3}\right]$ (log); $\left[10^{-4},5\times 10^{-2}\right]$ (log); $\left[10^{-4},10^{-1}\right]$ (log)
RF/Extra Trees (absolute and residual)	$n_{\mathrm{trees}}$, depth, min. leaf, max. features	{300,500,700}; {None,10,20,30}; {1,…,5}; {0.50,0.75,1.00}
XGBoost (absolute and residual)	$n_{\mathrm{trees}}$, depth, learning rate, subsample, column subsample, min. child weight, λ	{200,300,…,800}; {2,…,8}; [0.01,0.15] (log); [0.70,1]; [0.70,1]; [1,10]; $\left[10^{-3},10\right]$ (log)
GRU/LSTM controls	*h*, *p*, learning rate, weight decay, batch size, $\sigma_{\mathrm{AR}}$	{32,64,96}; {0,0.1,0.2}; $\left\{3,5,10\right\}\times 10^{-4}$; $\left\{10^{-5},10^{-4},10^{-3}\right\}$; {64,128,256}; {0,0.01,0.02}
TCN–ARX	channels *h*, *p*, learning rate, weight decay, batch size	{32,64,96}; {0,0.1,0.2}; $\left\{3,5,10\right\}\times 10^{-4}$; $\left\{10^{-5},10^{-4},10^{-3}\right\}$; {64,128,256}
Dual-branch fusion/GRAG-Net	$d_{x}$, $d_{y}$, $d_{g}$, *p*, learning rate, weight decay, batch size, $\lambda_{b}$, $\lambda_{g}$, $\sigma_{\mathrm{AR}}$	{32,64,96}; {16,32,64}; {32,64,96}; {0,0.1,0.2}; $\left\{3,5,10\right\}\times 10^{-4}$; $\left\{10^{-5},10^{-4},10^{-3}\right\}$; {64,128,256}; {0,0.025,0.05,0.10}; $\left\{0,10^{-4},10^{-3}\right\}$; {0,0.01,0.02}

**Table 5:** GRAG-Net configuration selected independently in all three chronological outer folds.

Hyperparameter/Setting	Selected Value
Process-history length $L_{x}$	64
Target history length $L_{y}$	16
Process hidden dimension $d_{x}$	96
Autoregressive hidden dimension $d_{y}$	64
Gate hidden dimension $d_{g}$	64
Dropout probability *p*	0
Learning rate	$5\times 10^{-4}$
Weight decay	$10^{-4}$
Batch size	64
Branch-loss weight $\lambda_{b}$	0.10
Gate-balance weight $\lambda_{g}$	$10^{-4}$
AR-noise standard deviation $\sigma_{\mathrm{AR}}$	0
Gate type	Dynamic
Residual reconstruction	Enabled
Branch-disagreement input $d_{t}$	Enabled
Process-change input $s_{t}$	Enabled

**Table 6:** Predictive performance across the three chronological outer folds and five final seeds. Values are descriptive mean ± standard deviation over 15 fold–seed evaluations per model. The three chronological folds, not the 15 repeated runs, are the primary independent evaluation periods; formal paired uncertainty is assessed separately by moving-block bootstrap. The best value is shown in bold and the second-best value is underlined. For MBD, ranking is based on absolute magnitude.

Model	MAE	RMSE	$R^{2}$	sMAPE (%)	MedAE	MBD (×10^−3^)	Pearson *R*
Persistence	0.00650±0.00120	0.01384±0.00123	0.93164±0.00171	4.219±1.035	0.002925±0.000721	$\underline{0.002\pm 0.074}$	0.96581±0.00088
Linear extrapolation	$\underline{0.00351\pm 0.00073}$	0.01221±0.00064	0.94636±0.00608	$\underline{2.582\pm 0.670}$	**0.00000001 ± 0.00000000**	**−0.00046 ± 0.00691**	0.97690±0.00271
SVR–ARX	0.00426±0.00082	0.01028±0.00069	0.96209±0.00306	3.234±0.731	0.001481±0.000442	0.175±0.250	0.98136±0.00178
Random Forest–ARX	0.03303±0.00865	0.04466±0.00835	0.24782±0.33249	21.721±6.979	0.024828±0.009408	−9.354±14.575	0.64258±0.17946
Extra Trees–ARX	0.03862±0.00547	0.05036±0.00583	0.07906±0.22073	24.658±4.844	0.030795±0.005488	−7.167±13.422	0.52925±0.19886
XGBoost–ARX	0.01673±0.00684	0.02558±0.00949	0.75924±0.13539	11.233±4.387	0.010690±0.004234	0.144±1.807	0.92815±0.02049
Residual SVR	0.00396±0.00086	0.01060±0.00059	0.95945±0.00638	2.863±0.786	0.001126±0.000403	−0.003±0.324	0.98075±0.00255
Residual RF	0.00771±0.00167	0.01458±0.00180	0.92173±0.02439	5.889±2.441	0.003865±0.001026	−1.971±3.542	0.96391±0.00714
Residual ET	0.00718±0.00097	0.01399±0.00091	0.92972±0.00666	4.850±0.981	0.003897±0.001175	−1.413±2.479	0.96624±0.00117
Residual XGBoost	0.00729±0.00117	0.01473±0.00172	0.92013±0.02416	4.908±1.110	0.003267±0.000470	−1.244±2.077	0.96041±0.01096
Process-only GRU	0.03460±0.01244	0.04377±0.01536	0.20871±0.56710	23.428±10.797	0.030827±0.013189	−6.764±28.431	0.72142±0.18620
AR–GRU	0.00453±0.00052	0.00979±0.00086	0.96555±0.00484	3.133±0.528	0.001856±0.000343	0.089±0.476	0.98290±0.00229
Joint GRU	0.00609±0.00043	0.01279±0.00089	0.93960±0.01466	4.003±0.409	0.002707±0.000479	−0.220±1.280	0.97004±0.00712
LSTM–ARX	0.00391±0.00075	$\underline{0.00889\pm 0.00097}$	$\underline{0.97123\pm 0.00720}$	2.785±0.676	0.001672±0.000750	0.706±1.027	$\underline{0.98642\pm 0.00282}$
TCN–ARX	0.00563±0.00131	0.01235±0.00187	0.94489±0.01330	3.742±1.029	0.002436±0.000695	0.104±0.307	0.97235±0.00701
Equal fusion	0.00408±0.00084	0.00941±0.00077	0.96815±0.00445	2.958±0.840	0.001669±0.001331	−0.793±1.676	0.98473±0.00172
Global gate	0.00390±0.00068	0.00908±0.00116	0.97029±0.00602	2.827±0.724	0.001501±0.000962	−0.858±1.197	0.98557±0.00290
GRAG-Net	**0.00315 ± 0.00044**	**0.00790 ± 0.00064**	**0.97753 ± 0.00331**	**2.258 ± 0.462**	$\underline{0.000907\pm 0.000257}$	−0.034±0.621	**0.98898 ± 0.00156**

**Table 7:** Fold-level mean performance over five final seeds for the principal sequence and fusion models. Errors are in mole%.

Model	Fold 1 MAE/RMSE	Fold 2 MAE/RMSE	Fold 3 MAE/RMSE
Persistence	0.005999/0.013024	0.005622/0.013229	0.007867/0.015258
AR–GRU	0.004429/0.009054	0.004028/0.009738	0.005137/0.010575
LSTM–ARX	0.003099/0.007812	0.004444/0.009583	0.004193/0.009268
TCN–ARX	0.004208/0.010101	0.005681/0.013079	0.007007/0.013860
Equal fusion	0.003245/0.008557	0.004701/0.009559	0.004302/0.010111
Global gate	0.003175/0.008208	0.004230/0.008938	0.004297/0.010100
GRAG-Net	0.002853/0.007103	0.002867/0.008128	0.003724/0.008472

## Data Availability

The industrial SRU dataset analyzed in this study is publicly available in Mendeley Data, Version 1, at https://doi.org/10.17632/6p865gztg6.1. A reproducibility package containing the source code used for the final reviewer experiments, chronological split definitions, preprocessing protocol, hyperparameter search spaces and selected configurations, epoch-selection records, final random seeds, fold–seed predictions, evaluation metrics, bootstrap outputs, ablation results, and interpretability-analysis files has been retained and is available from the corresponding author.
